# Resequencing 200 Flax Cultivated Accessions Identifies Candidate Genes Related to Seed Size and Weight and Reveals Signatures of Artificial Selection

**DOI:** 10.3389/fpls.2019.01682

**Published:** 2020-01-16

**Authors:** Dongliang Guo, Haixia Jiang, Wenliang Yan, Liangjie Yang, Jiali Ye, Yue Wang, Qingcheng Yan, Jiaxun Chen, Yanfang Gao, Lepeng Duan, Huiqing Liu, Liqiong Xie

**Affiliations:** ^1^ National Center of Melon Engineering and Technology, Molecular Breeding Laboratory, College of Life Science and Technology, Xinjiang University, Urumqi, China; ^2^ National Center for Soybean Improvement, Key Laboratory of Biology and Genetics and Breeding for Soybean, Ministry of Agriculture, State Key Laboratory for Crop Genetics and Germplasm Enhancement, Nanjing Agricultural University, Nanjing, China; ^3^ Herbal Medicine Innovation Research Center, Agricultural Bureau of Zhaosu County, Yili, China

**Keywords:** flax (*Linum usitatissimum* L.), domestication, genetic diversity, seed size, genome-wide association studies, selective sweeps

## Abstract

Seed size and weight are key traits determining crop yield, which often undergo strongly artificial selection during crop domestication. Although seed sizes differ significantly between oil flax and fiber flax, the genetic basis of morphological differences and artificial selection characteristics in seed size remains largely unclear. Here we re-sequenced 200 flax cultivated accessions to generate a genome variation map based on chromosome assembly reference genomes. We provide evidence that oil flax group is the ancestor of cultivated flax, and the oil-fiber dual purpose group (OF) is the evolutionary intermediate transition state between oil and fiber flax. Genome-wide association studies (GWAS) were combined with LD Heatmap to identify candidate regions related to seed size and weight, then candidate genes were screened based on detailed functional annotations and estimation of nucleotide polymorphism effects. Using this strategy, we obtained 13 candidate genes related to seed size and weight. Selective sweeps analysis indicates human-involved selection of small seeds during the oil to fiber flax transition. Our study shows the existence of elite alleles for seed size and weight in flax germplasm and provides molecular insights into approaches for further improvement.

## Introduction

Flax (*Linum usitatissimum* L.) has a worldwide distribution and is considered as an economically important crop in the world because of its vast fiber and seed oil (Fu, 2005; Cloutier et al., 2012). It has been suggested that cultivated flax were domesticated from a single domestication of wild flax (*Linum bienne*) (Allaby et al., 2005; Fu and Allaby, 2010) and has been used by humans for more than 10,000 years in the Near East (Austin, 2012). The difference in breeding selection during domestication contributed to two plant types with different agronomic traits for fiber flax and oil flax (Diederichsen and Ulrich, 2009). Typical oil flax has a shorter plant height, more branches and capsules, and larger seeds than those of the fiber flax varieties. However, as the genetic basis for phenotypic changes, the natural allelic variations responsible for flax varietal domestication-related morphological features have not been fully explored. Differences among individuals are stored in their genomes, which are reflected in the diversity of agronomic traits and differences in physiology and biochemistry (Lin et al., 2014). Finding the relationship between phenotype and genotype variation should help us understand the process of artificial selection and provides insights into further flax breeding improvement.

Seed size is one of the most important traits for crop yield. Large seed lines facilitate harvesting, processing and planting, which then leads to the selection of larger seed sizes during domestication (Tao et al., 2017). For instance, in rice, OsSPL13 positively regulates grain length and large-grain haplotypes are introgressed into tropical japonica varieties from indica varieties under artificial selection (Si et al., 2016). However, preferential selection for certain excellent phenotypes may result in small seed phenotypes being “bundled” together for artificial selection. For example, a SNP mutation of the *GL4* gene during African rice domestication resulted in the loss of seed shattering and smaller seeds than their wild ancestors. Selection of the loss of seed shattering allows small seeds to be selected together into African cultivated rice (Wu et al., 2017). Domestication of complex traits is likely to undergo different trade-offs according to the needs of breeders. Although seed sizes and weight differ significantly between oil flax and fiber flax, the genetic basis of morphological differences and artificial selection characteristics remain largely unclear.

Seed development is controlled by both genetic and external environment, which is a complex biological process. In angiosperms, the seed consists of seed coat, endosperm and embryo, which together determine the seed size (Bleckmann et al., 2014). In recent years, the regulation of seed development has attracted more and more attention due to the important role of seed related traits in agricultural production. Several functional genes controlling seed size have been reported, which are involved in plant hormones release, transcription factors, ubiquitin pathway components, P450 family members, G-protein components, microRNAs, genomic imprinting and other pathways. These genes control seed growth in the aspects of integument cell proliferation, embryo development, information exchange between seed coat, endosperm and embryo (Li and Li, 2016; Huang et al., 2017a). However, only SPX and EXS domain-containing protein (PHO1) has been reported as candidate genes for 1000-seed weight in flax (Xie et al., 2018a). The conservation of seed size regulators and pathway connectivity in different species may help us understand the seed development in flax.

Linkage mapping and association mapping are popular methods for the identification of underlying genetic variations in agronomic traits (Lin et al., 2014; Du et al., 2018). Linkage mapping uses biparental hybrids population such as F_2_ and recombinant inbred line (RILs), etc (Xu et al., 2015; Palanga et al., 2017). However, the limited genetic diversity of biparents and poor map resolution limit the efficiency of gene mining (Yano et al., 2016). Compared with linkage based-mapping, association mapping is based on existing natural or artificially constructed populations and has a wealth of diverse materials to choose (Huang et al., 2012b; Dell’Acqua et al., 2015). Most alleles in one locus of the mapping population can be examined, and genetic variation loci of many traits can be detected in a single study (Lipka et al., 2015). In theory, association mapping is more accurate than linkage mapping, and it is easier to achieve fine mapping, or even directly locate the gene itself (Yano et al., 2016; Du et al., 2018). As a powerful tool, association mapping has been successfully applied to detection of agronomic traits in numerous crop species (Lin et al., 2014; Zhou et al., 2015; Yano et al., 2016; Du et al., 2018).

During the past decades, genetic studies on flax agronomic traits based on association mapping have been reported. Soto-Cerda et al. (2014) used 464 SSR markers to map 9 agronomic traits in 390 flax germplasms, and identified 12 significant markers associated with 6 agronomic traits. 193 SSR markers were used to perform association analysis on 124 flax samples using GLM and MLM models, 32 loci were found to be associated with yield-related traits (Deng, 2013). Xie et al. performed genome-wide association studies (GWAS) on 5 yield-related traits of 224 flax samples using 34,932 SNPs based on GLM and MLM, and a total of 42 SNPs loci were identified and 15 candidate genes were obtained (Xie et al., 2018a). Subsequently, Xie et al. (2018b) performed GWAS on 13 agronomic traits using the same data set, only 6 traits were associated with 16 SNPs. In addition, a total of 17,288 SNPs were employed to perform GWAS for 11 traits in three different bi-parental flax mapping populations composed of 260 lines, and 33 quantitative trait loci (QTL) were detected (You et al., 2018a). Soto-Cerda et al. (2018) used 771,914 filtered SNPs to perform GWAS for mucilage (MC) and hull content (HC) in flax seeds, and seven and four QTLs were identified for MC and HC, respectively. Nevertheless, these studies were based on SSR and Reduced-Representation Genome Sequencing such as SLAF and GBS, which are far less efficient and accurate than re-sequencing.

In this study, we investigated 200 worldwide flax cultivars, representing a wider geographical distribution and a richer phenotypic variation. We performed more than 12× depth genome-wide resequencing based on chromosome assembly reference genomes and developed 1,179,402 informative SNP markers to estimate the genetic diversity and population structure. Subsequently, we attempted to identify candidate regions related to seed size and weight by GWAS combining with LD Heatmap. Thirteen candidate genes were finally obtained by screening candidate region variation sites and detailed gene annotation. We also detected the polymorphism patterns and characteristics in 13 candidate genes and revealed signatures of artificial selection. This genetic resource could contribute to the development of molecular markers and the breeding of flax varieties in future.

## Materials and Methods

### Plant Material

A total of 901 flax germplasms were collected worldwide to construct a germplasm resource bank. 541 accessions with wide geographical distribution and rich genetic diversity in phenotypic traits were selected to construct an original population. Then 200 accessions were used to construct a core collection for genome variation analysis and GWAS in this study. Among the 200 accessions, 87 were from the core collection of Plant Gene Resource Centre in Canada (PGRC), 105 from the United States National Plant Germplasm System (U.S.NPGS), and 8 from the Chinese Crop Germplasm Resources Information System (CGRIS) (**Supplementary Table S1**).

### Planting and Phenotyping

For phenotyping, the 200 accessions were planted in the experimental fields of Dali Economic Crop Research Institute (25°44′N, 101°52′E; altitude, 1,118.4m), Dali, Yunnan, China, in October of 2016 (2016DL) and Urumqi experimental base (43°55′N, 87°42′E; altitude, 688.0m), Urumqi, Xinjiang, China, in May of 2017 and 2019 (2017UR, 2019UR) as well as at the YiLi State experimental fields of the Institute of Agricultural Sciences (43°55′N, 81°23′E; altitude, 681.6m), YiLi, Xinjiang, China, in March of 2019 (2019YL). A randomized complete block design with three biological replicates was used for field experiments. Each cultivar was planted in five rows with a length of 2 m, and spacing of 25 cm between rows. The experimental area was surrounded by 1.5m wide isolation plot, and the field management was carried out in a conventional way. Seed length (SL), seed width (SW) and 1,000-seed weight (1,000-SW) were measured after harvest. The 1,000-seed weight was the weight of 1,000 mature, full and clean seeds counted by manual. High-resolution scanner was used to capture the image of seeds. Image J 1.8.0 assisted to accurate measure seed length and width manually by measuring the distances between the longitudinal axis and horizontal axis of twenty seeds, respectively. Three replicates were performed in the same environment.

### Sequencing and Genotype Calling

Flax seedlings were planted in greenhouse until 25-day-old. Genomic DNA was extracted from leaves with the plant genomic DNA extraction kit (TIANGEN). The qualified DNA samples were randomly fragmented by enzyme digestion and Covaris S2. The fragmented DNA was used for a DNA library construction with the NEBNext DNA Library Prep Reagent Set for Illumina (BioLabs). The DNA library was sequenced using an Illumina HiSeq X Ten (Illumina Co, Ltd.), and a total of 762.94 Gb of genomic sequence data were obtained. All reads were mapped against the reference genome (Wang et al., 2012; You et al., 2018b) (Chromosome assembly without annotated information) (https://www.ncbi.nlm.nih.gov/nuccore?LinkName=pubmed_nuccore&from_uid=22757964) using bwa-mem with the -M option of BWA software (Li and Durbin, 2009). The mapped reads were realigned using SAMTOOLS (Li et al., 2009), and the minimum allele frequency (MAF) of the single nucleotide polymorphisms (SNPs) was calculated using VCFtools software. After excluding the low-quality SNP, only high-quality SNPs (missing rate < 0.2, coverage depth ≥ 3, MAF ≥ 0.05 (674,074 SNPs) or MAF ≥ 0.01 (1,179,402 SNPs) were retained for subsequent analyses. SNPs with a MAF ≥ 0.01 were used for phylogenetic and population structure analyses, whereas SNPs with a MAF ≥ 0.05 were used in GWAS.

### Phylogenetic Tree and Population Structure

To reveal the phylogenetic relationship between the 200 flax cultivated accessions, a neighbor-joining tree was constructed using TreeBest v1.9.2 (Vilella et al., 2009) with 1,000 bootstrap replicates. Using the same data set, we also investigated the population structure using Admixture 1.23 (http://software.genetics.ucla.edu/admixture/index.html) and set the K value to 2~8. The most likely group number was determined according to the variation rule of an *ad hoc* statistic △K, and the genetic structure map was constructed. In addition, we performed principal-component analysis (PCA) using the software GCTA (Yang et al., 2011), and two-dimensional coordinate map was drawn according to the first two eigenvectors.

### Linkage Disequilibrium Analyses

The linkage disequilibrium (LD) between SNPs for the oil flax, oil-fiber dual purpose, and fiber flax accessions was evaluated using squared Pearson’s correlation coefficient (*r* ^2^) in the Haploview 4.20 software (Barrett et al., 2005) on the basis of 1,179,402 SNPs (MAF ≥ 0.01), with the Haploview parameters ‘ -pedfile -info -log –maxdistance 500 -minMAF 0.01 -dprime –memory 2000 ’. The LD decay was calculated based on *r* ^2^ and the distance between the two given SNPs. The average *r* ^2^ value was calculated for pairwise markers in a 500 kb window and averaged across the whole genome, and LD decay were drawn using an R script.

### Genome-Wide Association Study (GWAS)

In total, 674,074 high-quality SNPs (missing rate < 0.2, coverage depth ≥ 3, MAF ≥ 0.05) were used to perform GWAS for seed size and weight in 200 accessions. GWAS was performed using a mixed linear model (MLM) with the GEMMA software package (Kang et al., 2010). For the MLM analysis, we used the equation:

y=Xα+Sβ+Kµ+e

where *y* is a vector of phenotype; *X* represents genotype; *S* is a population structure matrix built up based on the top three PCs, and *K* is a kinship matrix built up based on simple matching coefficients calculated from the nucleotide polymorphisms; *α* and *β* represent fixed effects, and *μ* represents random effects; Where *e* is a vector of random residual effects. The significance thresholds (1/n) was set to control the genome-wide type I error rate of GWAS by modified Bonferroni correction(Guo et al., 2018); n represents the effective number of independent SNPs (Li et al., 2012). The *P*-value thresholds for significance in the flax population were approximately 1.0× 10^−5^.

### Significant SNP Annotation and Candidate Gene Screening

Rapid identification of new genes associated with agronomic traits by GWAS was performed as described previously (Yano et al., 2016) with slight modifications in this study. The process includes: (i) According to the GWAS associated loci, we constructed LD Heatmap surround Manhattan peak region using the R package “LD Heatmap” (Shin et al., 2006) to define candidate regions. (ii) For SNP in candidate regions, the log_10_(*P*) ≥ 4 mapping to flax reference genome (Wang et al., 2012) (Annotated information but chromosome not assembled) http://phytozome.jgi.doe.gov/pz/portal.html#!info?alias=OrgLusitatissimum) was performed for functional annotation. (iii) We classified all the SNPs in the candidate region into three groups. Group I included SNPs significantly associated with trait variation in the GWAS (−log_10_(*P*) ≥ 5), and located in the exon, intron and promoter regions. It should be reasonably expected that such polymorphism will have higher probability in functions variation or be more closely linked to genes. Group II included SNPs significantly associated with trait variation as group I and located on outside coding regions. Group III included SNPs not significantly associated with trait variation. (iv) Using Group I and Group II as a major and assistant screening site respectively, and combining with detailed gene function annotation, candidate genes were obtained. The SNP recombination within the candidate gene divides the varieties into distinct haplotypes, and we compare the phenotypic differences in the distinct haplotypes by non-parametric *t* test.

### Graphical Phenotype and Genotype Visualization

To display the relationship between population genetic structure and phenotypic distribution, we generated graphical phenotypes. Because the phenotypic data are distributed in different orders of magnitude, here we used zero-mean normalized phenotypes values of 7 major agronomic traits, including plant height (PH), technical length (TL), number of branches (BN) and capsules (CN), 1,000-seed weight (1,000-SW), seeds length (SL) and width (SW). To schematically display structural variation of candidate genes, we also generated graphical genotypes. In regions of candidate genes, SNPs were converted to numeric genotypes as 1, −1 and 0, which corresponded to major and minor polymorphisms and miss sites, respectively. Clustering the tested population was carried out using the hierarchical clustering algorithm based on candidate gene haplotypes, and the clustering information was used to generate graphic genotypes. In each polymorphic locus, the major and minor alleles were expressed in red and green, respectively, and the miss sites were represented in black. Phenotype and genotype visualization graphics conducted using the MeV4.9.0 software (Multi Experiment Viewer, https://sourceforge.net/projects/mev-tm4/files/mev-tm4/).

### Identification of Selective Sweep of Candidate Gene

Nucleotide diversity (π) is often used to measure the level of population diversity (Tajima, 1983; Lin et al., 2014). We used a 50 Kb sliding window size with a 25Kb step size to measure the level of nucleotide diversity in the genome regions in oil flax, fiber flax, and oil-fiber dual purpose flax. To test whether candidate genes for seed size and weight have undergone artificial selection, we selected all SNPs within candidate genes and calculated the level of nucleotide diversity at each locus in different populations using DnaSP 5.1 (Librado and Rozas, 2009). At the same time, we compared the distribution of allele frequencies of strong SNPs which were located in candidate genes. The large-seed and small-seed alleles were shown in purple and orange, respectively.

## Results

### Phenotypic and Genomic Variation

Abundant phenotypic variation is the basis for efficient genome wide association study (GWAS). The association mapping population in this study highly conserved the genetic diversity of the original population, and the three traits (SL, SW and 1,000-SW) showed abundant variations in 200 flax cultivated accessions (**Table 1**). Under four different environments, the maximum variation coefficient of 1,000-seed weight was 28.32%, and the weight range was from 2.94 to 10.50g. The variation coefficients of seed length and width were smaller, 10.05% and 9.66% respectively, and the seed length and width ranged from 3.11 to 5.42 mm and from 1.76 to 2.85 mm, respectively (**Table 1**). This suggested that the three seed size-related traits varied greatly, thereby providing a basis for the excavation of excellent alleles.

**Table 1:** Comparison of seed size and 1,000-seed weight variation between the 200 flax cultivated accessions used in this study and the 541 flax germplasm resources.

Environments	*n*	Traits	Maximum	Minimum	Range	Mean	*SD*	CV (%)
2016DL	541	1,000-SW	10.51	2.89	7.62	5.21	1.37	26.39
		SL	5.42	3.11	2.31	4.09	0.39	9.52
		SW	2.72	1.72	1.00	2.16	0.16	7.32
2016DL	200	1,000-SW	10.50	2.94	7.56	5.06	1.43	28.32
		SL	5.42	3.11	2.31	4.03	0.40	9.87
		SW	2.71	1.84	0.87	2.14	0.15	6.98
2017UR	200	1,000-SW	9.24	3.40	5.84	5.14	1.08	21.03
		SL	5.21	3.65	1.56	4.24	0.33	7.81
		SW	2.69	1.89	0.80	2.15	0.13	6.11
2019UR	200	1,000-SW	10.39	3.83	6.56	5.56	1.26	22.64
		SL	5.14	3.30	1.84	4.08	0.41	10.05
		SW	2.72	1.67	1.05	2.12	0.20	9.66
2019YL	200	1,000-SW	10.21	3.79	6.42	5.70	1.19	20.97
		SL	5.45	3.64	1.81	4.42	0.35	7.94
		SW	2.85	1.76	1.08	2.27	0.17	7.34

n, sample size; SD, standard deviation; CV (%), coefficient of variation.

Whole-genome resequencing was performed with the 200 flax association mapping population, and obtained a total of 762.94 Gb of sequences. The high quality clean data was 761.45 Gb, with an average 3.80 Gb for each accession. The average depth was 12.28× and covered 94.42% of the reference genome (chromosome assembly reference genomes) (You et al., 2018b). After aligning the reads against the flax reference genome, a set of 1,179,402 SNPs (missing rate < 0.2, coverage depth ≥ 3, MAF ≥ 0.01) were generated for subsequent analyses. Among them, 674,074 high-quality SNPs with MAF ≥ 0.05 were used to construct a high-density genome variations map (an average of 1.8 SNPs per kb) (**Supplementary Figure S1**) and for the genome-wide association studies of seed size and 1000-seed weight. This high-density SNP dataset serves as a new resource for flax biology and breeding.

### Population Structure and Linkage Disequilibrium

**Figure 1:**
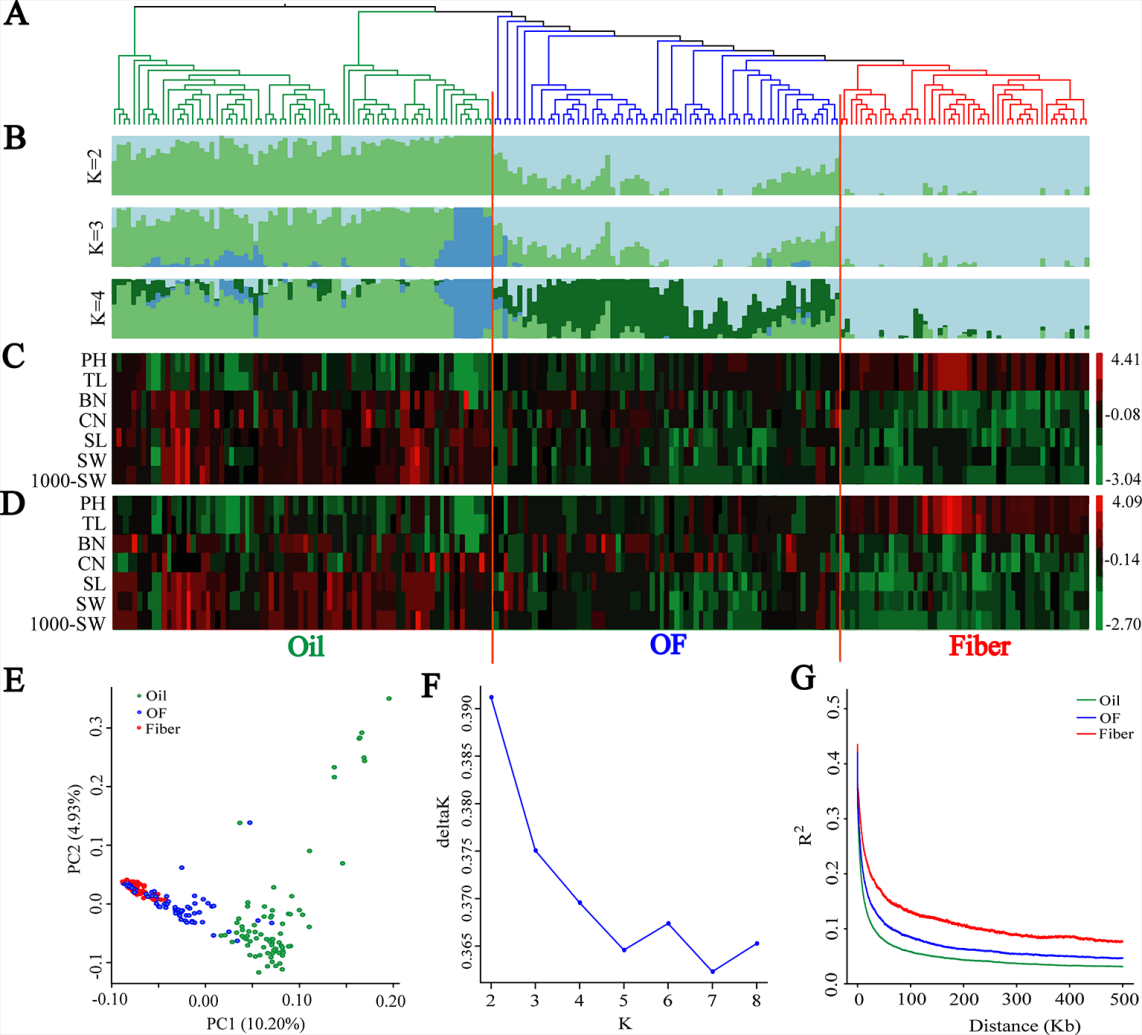
Population genetic structure and Linkage disequilibrium (LD) decay among 200 flax cultivars. **(A)** Neighbor-joining tree of 200 flax accessions was generated using 1,179,402 SNPs. **(B)** Population structure analysis with different numbers of clusters (K = 2, 3 and 4). **(C**, **D)** Graphical phenotype of 7 major agronomic traits. **(C)** 2016DL, **(D)** 2017UR. **(E)** PCA for the 200 flax varieties. **(F)** The most likely group number is determined using ad-hoc statistic △K for K values ranging from 2 to 8. **(G)** LD decay distance in three groups. Oil flax, oil-fiber dual purpose flax (OF), and fiber flax are drawn in green, blue and red colors, respectively.

A subset of 1,179,402 SNPs was used to construct neighbor-joining tree, and genetic population structure, and to carry out principal component analysis (PCA) to quantify the population structure and genetic relationships between the 200 flax cultivated accessions. The cultivated flax could be divided into oil flax, oil-fiber dual purpose (OF) flax and fiber flax groups (**Figures 1A, B**) that exhibited strong morphological distribution pattern, a result further supported by graphical zero mean normalized phenotype (**Figures 1C, D** and **Supplementary Figure S2**). In the PCA (**Figure 1E**), oil flax is independently distinguished, but the cross of fiber and OF groups indicates that the genetic relationship between fiber flax and OF flax is closer, but far from oil flax, a result further supported by neighbor-joining tree and genetic population structure (**Figures 1A, B**). Interestingly, a small subgroup in the oil flax groups was clearly distinguished from other oil cultivated varieties, and the genetic distance was farther than that between the fiber and OF groups (**Figure 1B**, K = 3). These varieties exhibited a dwarf phenotype, suggesting that plant height was an important selective trait in the early stage of oil flax breeding. We also found that some oil flax had fiber flax characteristics (**Figures 1B–D** and **Supplementary Figure S2**), which indicated that these varieties might be affected by the introgression or gene flow during the breeding process. Nucleotide diversity was higher in the oil group (*π* = 0.703 × 10^−3^) than in the OF group (*π* = 0.482 × 10^−3^) and the fiber group (*π* = 0.357 × 10^−3^). These results indicated that the oil flax group is the ancestor of cultivated flax, and the OF group is the evolutionary intermediate transition state between oil and fiber flax.

Population structure and linkage disequilibrium (LD) extent are two main factors affecting the correctness and accuracy of GWAS identification of candidate genes (Myles et al., 2009; Hamblin et al., 2011; Lipka et al., 2015). Diversified varieties usually represent a strong population structure, which creates false associations between phenotypes and unlinked markers during GWAS (Yano et al., 2016). So we determined the most likely group number using ad-hoc statistic △K for K values ranging from 2 to 8 (**Figure 1F**). △K shows the highest peak at k = 2. In addition, △K decreases sharply when K increases from 2 to 5, consequently, the most likely group number was at K = 2. This result is further supported by population structure and principal component analysis (**Figures 1B, E**). Another limitation was that large LD extent makes GWAS very difficult to finely map candidate genes. The LD decay with the increase of physical distance and varied among different populations (**Figure 1G**). The LD extent in oil flax was the lowest at about 6.4 kb, and in the OF and fiber groups LD increased to 11 kb and 25.3 kb, respectively. The LD extent in cultivated flax is much lower than that in cultivated rice (123 kb and 167 kb in *indica* and *japonica*, respectively) (Huang et al., 2010) and cultivated soybean (83 kb and 133 kb in landraces and improved cultivars, respectively) (Zhou et al., 2015), and even lower than those of LD extent in wild rice (*Oryza. rufipogon*, 20 kb) (Huang et al., 2012a) and wild soybean (*G. soja*, 27 kb) (Zhou et al., 2015). In the above studies, the LD extent (indicated by *r* ^2^) was measured as the physical distance at which the *r* ^2^ dropped to half of its maximum value, which was consistent with this study. This indicates that flax has obvious advantages in using LD compared with other crops, and the low LD extent makes it relatively easy to screen genes that regulate important agronomic traits.

### Genome-Wide Association Studies for Seed Size and 1,000-Seed Weight

The variation of flax seed shape is very small, and seed length, seed width and 1,000-seed weight were significantly positively correlated (**Supplementary Figure S3**). To identify genetic variation for seed size and weight in cultivated flax, we performed GWAS using a set of seed size-related traits (including SL, SW, 1000-SW). Manhattan plots and quantile–quantile plots for seed size-related traits from four environments are shown in **Supplementary Figures S4–S7**. The GWAS generated 599 significant association SNP loci in the four environments, including 289 SNPs in 2016DL, 158 SNPs in 2017UR, 80 SNPs in 2019UR, and 149 SNPs in 2019YL. The overlapping SNPs of SL, SW and 1,000-SW in each environment were 33, 11, 2, and 1, respectively. At the same time, 77 SNP loci were found in at least two environments. GWAS overlapping peaks for size-related traits and repetition in varied environments are focused on searching for genes that regulate seed size and weight.

**Figure 2:**
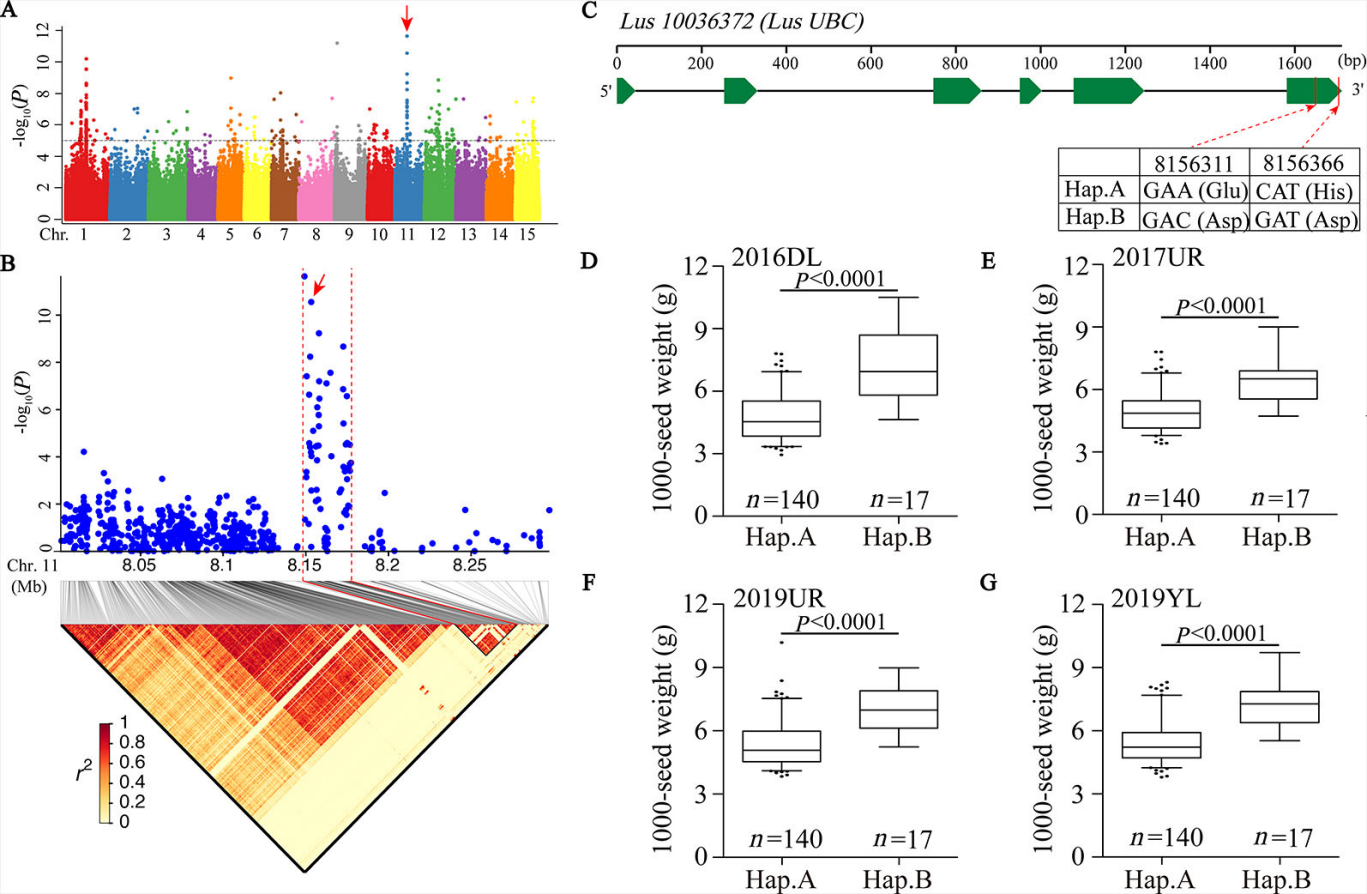
GWAS for seed size and 1000-seed weight traits, and candidate genes were obtained for the peak region on chromosome 11. **(A)** Manhattan plot for 1000-SW in 2016DL. The red arrow indicates the position of the strong peak on chromosome 11. The horizontal dashed line indicates the significance threshold (*P* = 1 × 10^−5^). **(B)** Local Manhattan plot (top) and LD heatmap (bottom) surrounding the peak on chromosome 11. The red arrow indicates a significant SNP in candidate gene *Lus10036372*. **(C)** Exon-intron structure of *Lus10036372* and haplotypes in that gene. (**D**–**G**) Boxplots for 1000-SW based on the haplotypes (Hap.) for *Lus10036372* in 2016DL **(D)**, 2017UR **(E)**, 2019UR **(F)** and 2019YL **(G)**. The difference between haplotypes was analyzed by non-parametric *t* tests.

We first focused on a strong signal peak of 2016DL-1000 SW on chromosome 11 (SNP 8148722, −log(*P*) = 11.64), overlapping with 2017UR-SL, 2016DL-SL, 2016DL-SW and 2019YL-SL peaks (**Figure 2A**; **Table 2** and **Supplementary Figures S4, S5A**, and **S7A**). We predicted that the candidate regions ranged from 8.15 to 8.18Mb (28.10 kb) and contained 75 SNPs, of which 9 SNPs belonged to Group I and were located in four genes (**Figure 2B** and **Supplementary Table S2**). One of these genes, *Lus10036372*, is annotated as the ubiquitin-conjugating enzyme E2 (*LusUBC*) (**Supplementary Table S2**). Its homologous gene *UBC22* (**Supplementary Table S3**) has been previously reported to control the seed size and weight in *Arabidopsis* (Xu, 2014). This gene contains 6 SNPs, three of which locate in exon and two SNPs induces nonsynonymous mutations to form haplotype A and haplotype B. The SNP at position 8156311 (A/C) resulted in an amino acid change from Glu to Asp, and the SNP at position 8156366 (C/G) caused an amino acid change from His to Asp (**Figure 2C**).Varieties carrying haplotype B showed longer seed length and width and larger 1,000-seed weight than the varieties carrying haplotype A (**Figures 2D–G** and **Supplementary Figure S8**). These results supported that *Lus10036372* could be a candidate gene of overlapping peak signal on chromosome 11.

**Table 2:** A subset of associated SNPs and seed size and 1000-seed weight candidate genes based on genome-wide association studies.

Environment and traits^a^	Chr^b^	Position	Scaffold^d^	Location^e^	–log_10_ *P*	Candidate genes	Annotation
2016DL-1,000 SW	1	13675535^c^	scaffold61	259597	7.36	*Lus10008438*	Kinesin family (KF)
2017UR-1,000 SW	1	13675535^c^	scaffold61	259597	6.03		
2016DL-SW	1	13675535^c^	scaffold61	259597	5.68		
2016DL-SL	1	13677254^c^	scaffold61	257878	5.94		
2019YL-SL	4	8904836^c^	scaffold376	199412	5.15	*Lus10029035*	Cytochrome P450 (P450-1)
2017UR-SW	4	8917679	scaffold376	212255	5.35		
2019YL-SL	4	13877858^c^	scaffold310	987727	5.15	*Lus10034378*	Ribosomal protein (RP)
2019YL-1,000 SW	4	13884212	scaffold310	981373	5.48		
2019UR-1,000 SW	4	13884281	scaffold310	981304	5.43		
2017UR-1,000 SW	5	8559897^c^	scaffold568	455577	7.80	*Lus10011772*	Malate dehydrogenase (MDH)
2017UR-SW	5	8559897^c^	scaffold568	455577	6.66		
2016DL-1,000 SW	5	8582808	scaffold568	432666	8.97		
2016DL-SW	5	8582808	scaffold568	432666	6.44		
2017UR-SW	6	6431359	scaffold212	652035	5.43	*Lus10017775*	Cytochrome P450 (P450-2)
2016DL-1,000 SW	6	6433163	scaffold212	653839	6.47		
2016DL-1,000 SW	7	6015848	scaffold151	1361917	8.03	*Lus10035470*	26S proteasome regulatory subunit (RPN)
2016DL-SW	7	6015848	scaffold151	1361917	5.84		
2017UR-1,000 SW	7	6011047^c^	scaffold151	1357116	6.01		
2019UR-1,000 SW	9	1710055^c^	scaffold1486	167586	5.70	*Lus10008949*	Ubiquitin carboxyl-terminal hydrolase (UCH-1)
2019YL-1,000 SW	9	1710055^c^	scaffold1486	167586	5.31		
2016DL-1,000 SW	9	1712440^c^	scaffold1486	169971	5.87		
2016DL-SW	9	1712440^c^	scaffold1486	169971	5.45		
2016DL-SL	11	8148722	scaffold57	339477	6.57	*Lus10036372*	Ubiquitin-conjugating enzyme E2 (UBC)
2017UR-SL	11	8150087	scaffold57	338112	5.61		
2016DL-SW	11	8152152	scaffold57	334085	5.12		
2016DL-1,000 SW	11	8152763^c^	scaffold57	335436	10.55		
2019YL-SL	11	8157465	scaffold57	330734	5.05		
2017UR-SW	12	16347940^c^	scaffold25	1315647	4.29	*Lus10043126*	Ubiquitin carboxyl-terminal hydrolase (UCH-2)
2017UR-1,000 SW	12	16347940^c^	scaffold25	1315647	4.51		
2017UR-SL	12	16347940^c^	scaffold25	1315647	7.07		
2019UR-1,000 SW	14	4549028^c^	scaffold1322	4509	5.87	*Lus10000489*	Ankyrin-repeat protein (ARP)
2019YL-1,000 SW	14	4549712^c^	scaffold1322	5193	6.01		
2017UR-SL	14	4561458	scaffold1322	16939	5.70		
2019YL-1,000 SW	14	6548762	scaffold359	445524	5.12	*Lus10013178*	COP1-interacting protein (CIP)
2017UR-SW	14	6570017^c^	scaffold359	424269	5.10		
2019UR-1,000 SW	14	6591565	scaffold359	402721	5.10		
2019UR-1,000 SW	14	7321617	scaffold115	43090	5.13	*Lus10004079*	Auxin canalization (AC)
2019YL-1,000 SW	14	7345452^c^	scaffold115	19255	6.08		
2019YL-SL	14	7363182	scaffold115	1525	5.95		
2019YL-SW	14	7363182	scaffold115	1525	5.06		
2017UR-SW	15	11940681	scaffold465	802457	5.79	*Lus10022567*	RING/U-box protein (RING/U-box)
2019YL-SW	15	11941273	scaffold465	801865	5.05		
2016DL-1,000 SW	15	11941703	scaffold465	801435	6.22		
2019UR-SL	15	11950997^c^	scaffold465	792141	5.75		
2017UR-SL	15	11950997^c^	scaffold465	792141	6.93		
2019UR-SW	15	11960489	scaffold465	782649	5.02		
2017UR-1,000 SW	15	11960489	scaffold465	782649	5.90		
2019UR-1,000 SW	15	11962968	scaffold465	780170	6.10		
2016DL-SW	15	11963946	scaffold465	778896	5.03		

a Correspondence of environment and traits, each block represents an overlapping peak of different environment and traits. ^b^Chromosome. ^c^SNP is located in exon, intron or promoter region of candidate gene. ^d,e^represent the position of SNP in the annotated genome and correspond to the chromosome.

Similarly, we analyzed an overlapping peak on chromosome 9 (**Table 2** and **Supplementary Figures S4B, C, S6C**, and **S7C**). The candidate region was predicted to map from 1.71 to 1.73 Mb (19.53K), which contained 91 SNPs (**Supplementary Figure S9A**). There were 6 SNPs to Group I, and these located in three genes. Among these genes, *Lus10008949* (*LusUCH-1*), which showed the highest -log_10_(*P*) value (SNP 1710055, -log(*P*) = 5.70), encoded a ubiquitin carboxyl-terminal hydrolase (USP7, UBP15) (**Supplementary Table S4**). In *Arabidopsis*, it was reported that the deubiquitinase UBP15 (ubiquitin -specific protease 15) regulates seed size by promoting cell proliferation (Du et al., 2014). This gene contains 36 SNPs, and only two SNPs induced nonsynonymous mutations to form four haplotype A-D. The SNP at position 1708112 (G/T) caused an amino acid change from Gin to His, and the SNP at position 1714014 (C/T) resulted in an amino acid substitution from Pro to Ser (**Supplementary Figure S9B**). The varieties carrying haplotype A had significantly shorter seed length and width, and smaller 1000-seed weight than did those with haplotype B (**Supplementary Figure S10**). These results supported that *Lus10008949* was a candidate gene on chromosome 9. Using the same strategy, we obtained 12 candidate genes on chromosomes 1, 4, 5, 6, 7, 9, 11, 14, and 15 (**Table 2**).

### Allele Heterogeneity Reduces the Intensity of Association Signals

**Figure 3:**
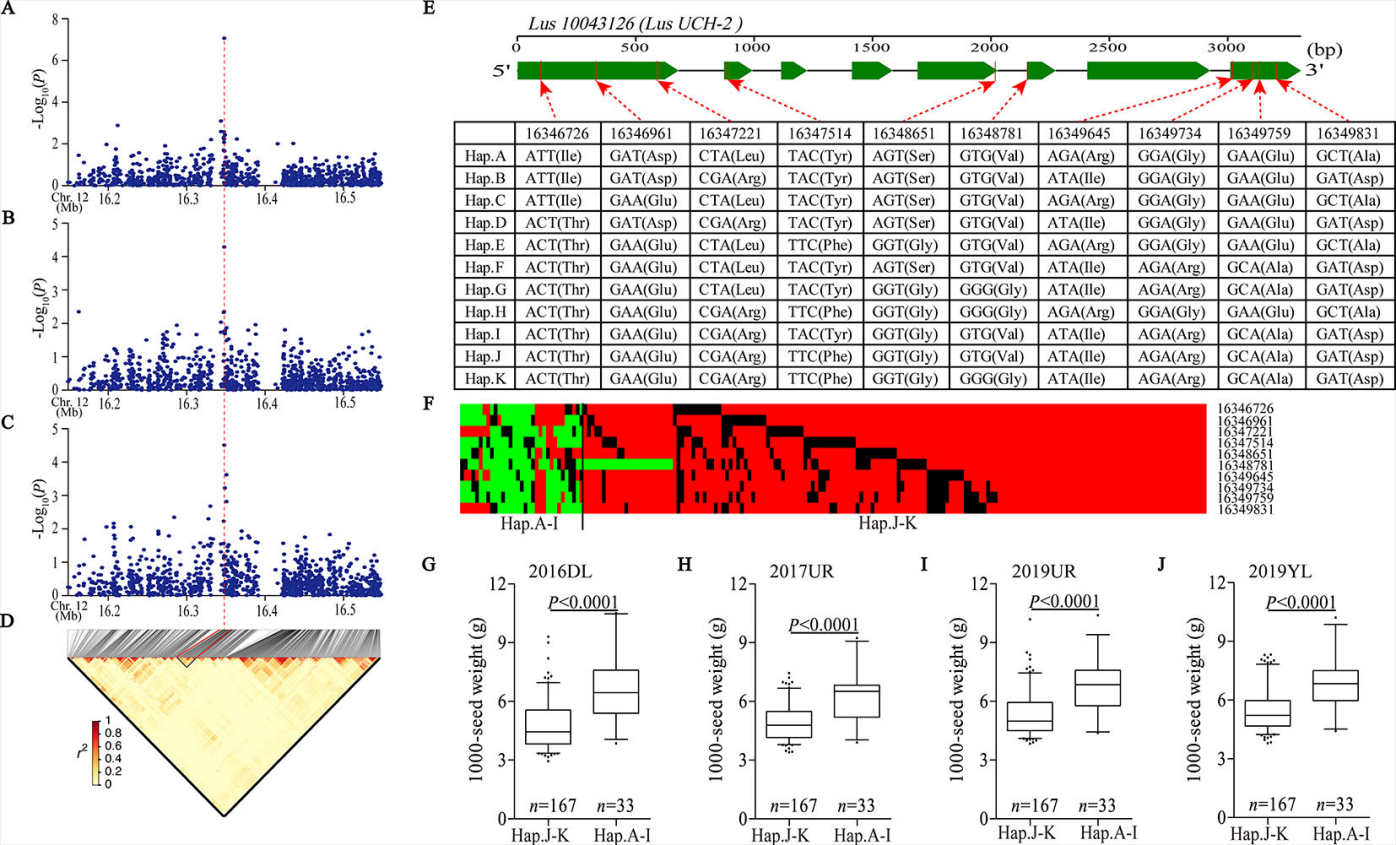
Analyses of the peak for seed size and 1,000-seed weight on chromosome 12. **(A**–**C)** Local Manhattan plot for SL **(A)**, SW **(B)**, and 1,000-SW **(C)** in 2017UR and LD heatmap **(D)** surrounding the peak on chromosome 12. Red dotted line indicates that the highest peak (SNP 16347940) on **(A**–**C)** and located in *Lus10043126*. **(E)** Exon–intron structure of *Lus10043126* and DNA polymorphism in this gene. **(F)** A schematic map of structural variation of *Lus10043126* was presented. The major and minor alleles at each polymorphic locus were represented in red and green, respectively, and the miss sites were represented in black. **(G**–**J)** Boxplots for 1000-SW based on *Lus10043126* function as follows: haplotypes A–I (functional alleles) and haplotypes j-K (nonfunctional alleles) in 2016DL **(G)**, 2017UR **(H)**, 2019 UR **(I)** and 2019YL **(J)**. The difference between haplotypes was analyzed by non-parametric *t* tests.

We detected a significant association signal (SNP 16347940, −log_10_(*P*) = 7.07) of 2017UR-SL on chromosome 12, which was also detected on 2017UR-SW and 2017UR-1000 SW and showed a weak association signal (4.51 and 4.29, respectively) (**Figures 3A–C**, **Table 2**). We estimated that the candidate region was 16.34 to 16.36 Mb (12.79K), which contains 50 SNPs, but was the only one that belongs to Group I and locates in *Lus10043126* (**Figure 3D** and **Supplementary Table S5**). This gene was annotated as ubiquitin carboxyl-terminal hydrolase (*LusUCH-2*). Here we investigated why this peak was weak and had only one SNP support. We focused on the local LD heatmap surrounding the peak, which showed a week linkage (**Figure 3D**). We assumed that the lack of markers support was due to the insufficient number of SNPs in the candidate region. However, we detected 20 SNPs in the candidate gene *Lus10043126*, and 17 SNPs located in exons. This means that it was unrelated to the number of markers. Ten SNPs induced nonsynonymous mutations to form 11 haplotypes A–K (**Figure 3E**). A single gene multiple variations result in allele heterogeneity, which make it difficult to show statistical significance because the effect of one SNP variation in a candidate gene can be compensated by the effects of other variations in the same gene. This may indicate that allele heterogeneity reduces the intensity of the associated signal.

Next, we constructed a schematic map of the structural variation of candidate genes. The major and minor alleles at each SNP locus were expressed in red and green, respectively, and the miss sites were represented in black (**Figure 3F**). We classified these cultivars into two groups, including haplotypes A–I (functional alleles) and haplotypes J–K (nonfunctional alleles). We compared the phenotypic differences between haplotypes A–I and haplotypes J–K, which clearly indicated that the cultivars carrying functional alleles (haplotypes A–I) showed longer seed length and width, and larger 1,000-seed weight than those carrying non-functional alleles (haplotypes J–K) (**Figures 3G–J** and **Supplementary Figure S11**). Finally, we suggested that the *Lus10043126* was a candidate gene of seed size on chromosome 12.

### Selective Sweep Signals in Candidate Genes of Seed Size and 1000-Seed Weight

The cultivated flax could be divided into oil flax, oil-fiber dual purpose (OF) and fiber flax groups with obvious morphological differences (**Figures 1A–D** and **Supplementary Figure S2**). In terms of seed size traits, there were significant differences among three groups. Oil flax has larger seed size and 1,000-seed weight, while fiber flax has smaller seed size and lighter seed weight, and OF flax is in the middle phenotype (**Supplementary Figure S12**). This result suggests that seed size may be preferred selection in different groups.

**Figure 4:**
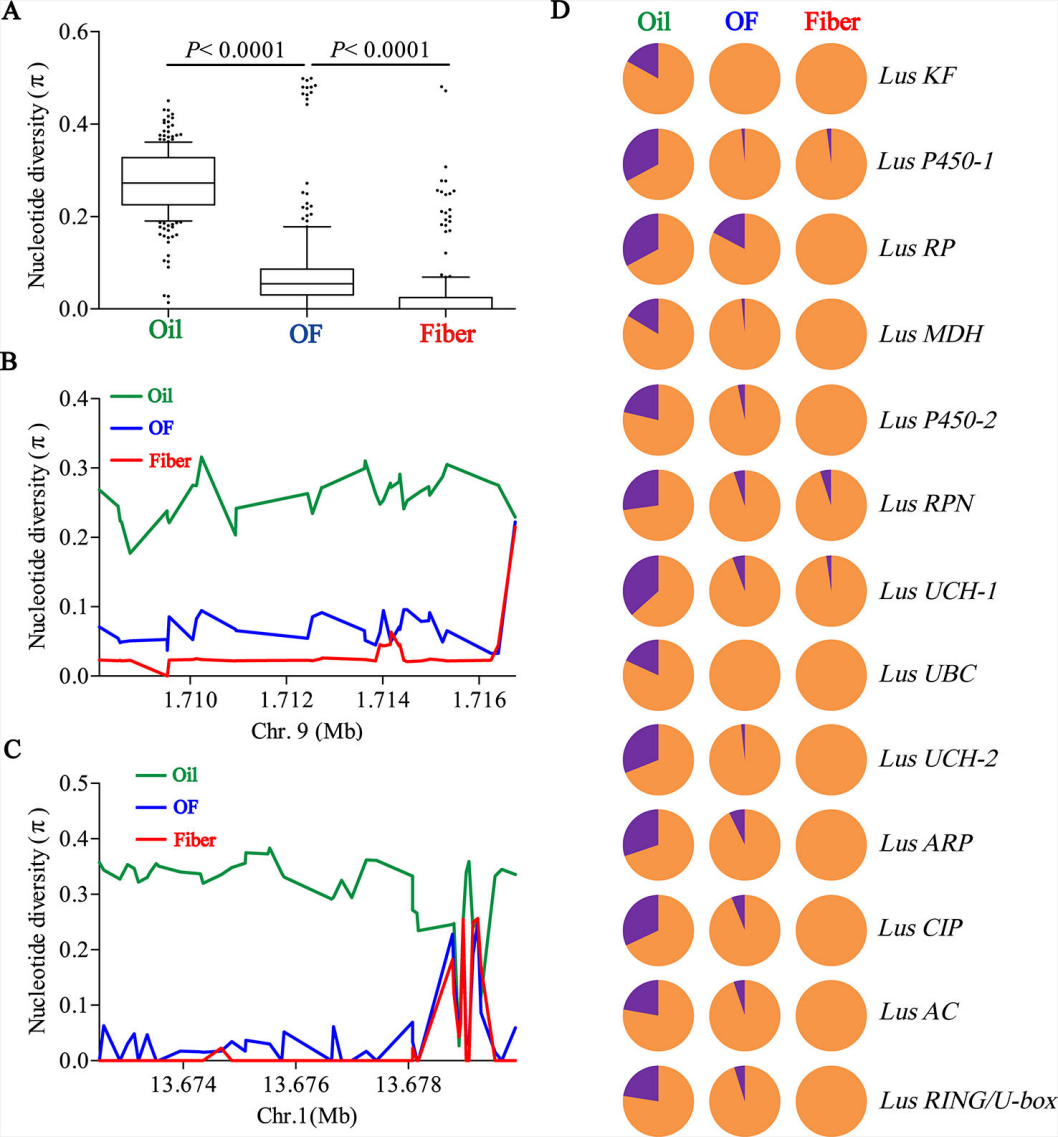
Distribution of nucleotide diversity (π) within candidate genes and allele frequency differences among Oil, OF and Fiber groups. **(A)** Boxplots for nucleotide diversity in seed size candidate genes among Oil, OF and Fiber groups. The difference was analyzed by two-tailed Student’s t tests. **(B**, **C)** Local nucleotide diversity (π) was distributed around *Lus10008949* on chromosome 9 **(B)** and *Lus10008438* on chromosome 1 **(C)** among Oil (green), OF (blue) and Fiber (red) groups. **(D)** The distribution of allele frequencies of strong SNPs are located in candidate genes were distributed in Oil, OF and Fiber groups. The large-seed and small-seed alleles are shown in purple and orange, respectively.

To determine whether seed size was selected artificially during flax domestication, we collated all SNPs loci in 13 seed size candidate genes and compared the differences of nucleotide diversity (π) among oil flax, OF flax and fiber flax. The diversity of candidate genes was significantly different among three groups (*P* < 0.0001, t test), and the highest diversity was found in oil groups and the lowest in fiber groups (**Figure 4A**). This result further supports the selection of seed size in flax breeding. Subsequently, we focused on two candidate genes, *Lus10008949* and *Lus10008438*, for which they contain more SNPs (36 SNPs and 45 SNPs, respectively). The whole region of *Lus10008949* showed strong selective sweeps, while the local area of *Lus10008438* showed obvious selective sweeps (**Figures 4B, C**).

Next, we detected allele frequencies of 13 candidate genes in three groups. The cultivars carrying large-seed alleles were mainly concentrated in oil groups, and a few were found in OF groups and rare in fiber groups (**Figure 4D**), which was consistent with the distribution of phenotypes in different groups (**Figures 1C, D**; **Supplementary Figures S2, S12**). These results indicated that small seeds were mainly selected in the breeding process during oil flax to OF flax, and small seeds were further selected during the domestication transition from OF flax to fiber flax. Large-seed alleles were mainly concentrated in oil groups, suggesting that large seed may be a major selection trait during oil flax improvement (**Figure 4D**). We tested the nucleotide diversity (π) between landrace and improved oil flax cultivar. But unexpectedly, we did not find the selective sweep region within the candidate gene during the landrace to improved oil cultivars (**Supplementary Figure S13**).

## Discussion

Abundant diversity in crop germplasm resources contains rich natural variations which facilitate the identification of elite alleles and crop improvement. Flax is one of the most important fiber and oil crop (Fu, 2005; Cloutier et al., 2012), although its yield is lower than other crops. By genome-wide association study in this study, we detected hundreds of significant SNPs and predicted 13 candidate genes associated with seed size. These elite alleles in germplasm may provide more genetic information for further flax improvement.

### Low LD Extent Achieves Fine Mapping Demanding Higher Marker Density

Association mappings based LD have provided high resolution for exploring genes that may sustain variability in phenotypes. The extent of LD reflects the requirement of marker density and mapping resolution in GWAS (Soto-Cerda et al., 2018). The LD extent between molecular markers is mostly influenced by recombination rate, genetic isolation, population sub-structure, selection effect, and other random factors, and the mating system of species also has a decisive influence on the LD extent (Rafalski and Morgante, 2004). Generally, LD extent is higher in self-pollinating plants and often ranges over several hundred kilobases as in rice and soybean (Yano et al., 2016; Zhou et al., 2015). However, in outcrossing plants such as maize, the LD extent is relatively low (Hufford et al., 2012). Interestingly, as a strictly self-pollinating crop, flax has a LD extent only ~15 kb in our study, which is consistent with the situation in *Arabidopsis* (Cao et al., 2011). Low extent of LD in flax makes it relatively easy to identify causal genes because a single LD block contains fewer genes. However, the low extent of LD in flax genome requires a higher marker density, and it needs a sufficient number of markers to cover each LD block in the whole genome. In addition, when SNPs were detected at very low depths (~0.013× to ~0.33×), sequencing errors could lead to the presence of minority SNPs (Lu et al., 2017). With the increase of sequencing depth (~10×), SNPs can be genotyped accurately and used directly for association and/or linkage analysis (Zhou et al., 2015). Therefore, in this study, we performed genome-wide resequencing over 12× depth to obtain 674,074 SNPs for GWAS to meet genome-wide detection well.

### Oil Flax Population is the Ancestor of Cultivated Flax

Molecular markers such as RAPD, AFLP, ISSR, SSR, IRAP, and SNP have been widely used in flax to measure the genetic diversity and population structure in different breeding programs (Soto-Cerda et al., 2012; Xie et al., 2018b), which provided the evinces to support the associations between geographic origin and morphological differences during flax seed spreading over the world (Smykal et al., 2011; Soto-Cerda et al., 2013; Chandrawati et al., 2017; Xie et al., 2018b). In the present study, cultivated flax was divided into oil flax, oil-fiber dual purpose (OF) and fiber flax groups. The population genetic structure is basically consistent with the morphological differences of cultivars. △K shows that the most likely group number is K = 2, which is consistent with previous studies (Soto-Cerda et al., 2018). Oil flax is basically clustered into one group and has a long genetic distance from OF and fiber groups (**Figures 1B, E**). Nucleotide diversity of oil groups (*π* = 0.703 × 10^−3^) was higher than that of OF (*π* = 0.482 × 10^−3^) and fiber groups (*π* = 0.357 × 10^−3^), and LD extent was lower than that of OF and fiber groups (**Figure 1G**), which is consistent with previous studies (Xie et al., 2018b). Overall, these morphological and genomic data support that the oil flax population is the ancestor of cultivated flax.

### Human-Involved Selection of Small Seeds During the Oil to Fiber Flax Transition

Seed size and weight play an important role in crop yield. Large-seeded genotypes often undergo intensive artificial selection and evolve into cultivated varieties with much larger seed size and weight than their wild progenitors (Tao et al., 2017), as demonstrated in rice, maize and soybean (Lu et al., 2013; Hirsch et al., 2014; Zhou et al., 2015). But interestingly, small seeds undergo artificially effective directional selection during the domestication transition from oil flax to fiber flax (**Figure 4**). Vegetative growth and reproductive growth are dynamically balanced processes, overly vegetative growth often results in phenotype of lower seed yield. For example, silage maize pays more attention to plant height and dry matter yield, and its seed yield is far less than that of dwarf elite maize, and crop breeding often increases seed yield by reducing plant height (Asano et al., 2011; Melchinger et al., 2016). Genetic antagonism effect between traits implies that some traits need to be “sacrificed” when some excellent phenotypes was artificially selected. Fiber flax breeding pays more attention to plant height because it is directly related to fiber yield (Zhang et al., 2018). Selection of plant height may result in small seed phenotypes being “bundled” together for artificial selection.

Oil flax showed larger seed size and 1,000-seed weight than fiber and dual-purpose flax, but we did not find any selective sweep signal in candidate genes during the landrace to improved oil flax cultivars (**Figures 1C, D**; **Supplementary Figure S2, S12**, and **S13**). There might be two possibilities: First, the mutation genes for seed size have not been effectively spread into the domestication process due to geographical restrictions (Liu and Zheng, 2017). Farmers may choose to obtain different loci mutation in geographically distinct region, which results in regionalized distribution of beneficial mutation accumulation, and increases the genetic diversity of these mutation loci; Second, the seed yield is controlled by many traits, such as branch number, capsule number, 1,000-seed weight, and so on. Small seeds can compensate for yield loss by increasing the seeds number (Moles et al., 2004). The necessity of seed size for controlling yield was reduced, and the selection pressure was decreased.

### GWAS of Seed Size and Weight Traits Reveal That Ubiquitin-Proteasome Pathway is Widely Involved in Seed Size Regulation in Flax

In this study, we obtained 13 candidate genes related to seed size and weight, of which the annotations of 6 genes were related to ubiquitin-proteasome pathway. It is very exciting because the ubiquitin-proteasome pathway is thought to be widely involved in the regulation of seed size and weight (Li and Li, 2014). Multiple components of the ubiquitin proteasome pathway were reported to regulate seed size and weight in numerous crops, however, to our knowledge, it has not been reported that multiple key genes in the whole pathway regulate seed size in a single study. A significant SNP peak (SNP 8148722, −log_10_(*P*) = 11.64) was detected in the *LusUBC* locus (*Lus10036372*) on chromosome 11, which encodes the ubiquitin-conjugating enzyme E2 (UBC) (**Figures 2A–C**). UBC is a carrier of ubiquitin transfer between ubiquitin activation enzyme E1 and ubiquitin ligase E3 (Sharma and Bhatt, 2017). In *Arabidopsis*, the knockout mutant plant of ubiquitin-conjugating enzyme (UBC22) significantly reduced the silique length and the seeds number, and nearly 90% of ovule abortion (Wang et al., 2016). The *Arabidopsis* null UBC22 mutant produced larger plants and seeds size, heavier seeds, and stored more protein and fatty acids (Xu, 2014). So in our opinion *Lus10036372* is a reliable candidate gene for this QTL region.

We detected nine environment and traits that showed overlap on chromosomes 15 (**Table 2**; **Supplementary Figures S4–S7**), and candidate genes were annotated as RING/U-box protein (*Lus10022567*). RING/U-box protein as E3 ubiquitin ligases can transfer ubiquitin from E2 to specific substrate proteins and has been reported to play an important role in plant morphology, hormone signaling and stress response (Cho et al., 2008; Qin et al., 2008; Plechanovova´ et al., 2012; Zhang et al., 2015; Wang et al., 2017). The RING containing E3 ligases EOD1 and DA2 were reported to negatively regulate seed size in *Arabidopsis* (Li et al., 2008; Xia et al., 2013). In rice, *GW2* encodes a RING-type E3 ligase. Loss-of-function *GW2* mutants showed an significantly increased cells number, an accelerated grain milk filling rate and a wider spikelet hulls, grain width, weight and yield (Song et al., 2007; Choi et al., 2018). Similar functions have also been reported in wheat and maize (Li et al., 2010; Su et al., 2011). Candidate genes *Lus10013178* were annotated as COP1-interacting protein (CIP). In *Arabidopsis,* CIP8 as a ubiquitin E3 ligase (Hardtke et al., 2002). This means that *Lus CIP* may play the role in ubiquitin E3 ligase for regulating seed size in flax.

Interestingly, we detected two candidate genes (*Lus10008949* and *Lus10043126*) in the peak regions on chromosomes 9 and 12 that were annotated as ubiquitin carboxyl-terminal hydrolase (UCH) (**Table 2**). UCH catalyzes the hydrolysis of ubiquitin carboxyl terminal polypeptide chain links, reversing regulation of protein degradation. In *Arabidopsis*, *SOD2* encodes a deubiquitinase UBP15 (ubiquitin -specific protease 15), which regulates seed size by promoting cell proliferation (Du et al., 2014). It should be noted that while a single mutant forms small seeds and organs, overexpression of *SOD2* results in seed and plant enlargement. *WIDE AND THICK GRAIN 1* (*WTG1*) encodes an otubain-like protease with deubiquitination activity. *Wtg1-1* mutant showed wide, thick, short and heavy grains and overexpression of *wtg1* resulted in narrow, thin and long grains by regulating cell expansion influences grain size and shape in rice (Huang et al., 2017b). These reports suggest that deubiquitination plays an important role in flax seed size regulation.

We detected *LusRPN* locus (*Lus10035470*) on chromosome 7, which encodes 26S proteasome regulatory subunit (**Table 2**). 26S proteasome is the main molecular machine responsible for protein degradation in eukaryotic cells. In *Arabidopsis*, the 26S proteasome regulatory particle subunit mutants exhibited many morphological defects, including abnormal embryogenesis, delayed growth rate, dwarfing phenotype and reduced stamen number or infertility (Smalle et al., 2003; Book et al., 2009). Functional deletion mutation subunit regulated particle RPT2a decreased the activity of 26S proteasome. The mutant increased cell expansion and compensated for the decrease of cell number, resulting in the enlargement of plant organs such as seeds and embryos (Kurepa et al., 2009). These candidate genes involve different components of ubiquitin-proteasome pathway that may regulate flax seed size through a complex interactions network.

### Scanning the Genome Provides Molecular Insights for Efficient Combination of Superior Parents

Seed size and weight are one of the most important components of crop yield, which often undergo strongly artificial selection during crop domestication (Tao et al., 2017; Wu et al., 2017). Although the seed size and weight are affected by environmental factors, it is mainly controlled by the genotype of maternal sporophytic and zygotic tissues (Jiang et al., 2013; Li and Li, 2015). Yield-related traits are often regulated by multiple genes. In particular, the genetic architecture of seed size is more complex, involving more than 400 genes (Huang et al., 2013; Wu et al., 2017). In this study, 599 SNPs (many overlap in effect) that were significantly related to seed size and weight were produced in GWAS, and QTLs were found on almost every chromosome (**Supplementary Figures S4–S7**). In fact, it may be very difficult to assemble multiple elite genes from different parents into a single variety through pyramiding breeding. However, introgression between different flax varieties may contribute to the accumulation of superior alleles during domestication, which may be a shortcut to speed up yield improvement. In our study, we found that flax varieties contained different large seed haplotypes, and the seed size and 1000-seed weight increased with the number of large seed haplotypes accumulation in flax varieties (**Supplementary Figure S14**). Fortunately, the line CIli 2719 showed the characteristics that it contained 10 large-seed haplotypes and the 1000-seed weight (~10g) was the largest in all germplasms in this study (**Supplementary Figure S14**). This means that line CIli 2719 as a parent to improve flax seed yield can greatly shorten the breeding period. It suggested that functional haplotypes scanning in variety is necessary before parent’s choice in complex trait breeding program. Choosing germplasms with much effective alleles or haplotypes may accelerate the process of pyramiding breeding through MAS. It may be a great potential strategy to improve the seed size and yield in flax.

## Conclusion

In the present study, we re-sequenced 200 flax cultivated accessions to generate a genome variations map containing 674,074 SNPs with an MAF ≥ 0.05. The genome-wide variation revealed that oil flax group is the ancestor of cultivated flax, and the oil-fiber dual purpose group is the evolutionary intermediate transition state between oil and fiber flax. We obtained 13 candidate genes related to seed size and weight, including 6 genes involved in the ubiquitin-proteasome pathway. Candidate gene selective sweep signals indicate human-involved selection of seed size during flax domestication. Fiber flax pays more attention to the selection of plant height, which may lead to small seed phenotype “bundling” together for artificial selection. This will improve our understanding of the genetic basis of seed size differences between fiber flax and oil flax and provide insights into how humans participation in the selection of seed size in flax domestication.

## Data Availability Statement

All sequencing data generated for this study can be found in the NCBI using accession number PRJNA590636 (ncbi.nlm.nih.gov/bioproject/PRJNA590636).

## Author Contributions

DG, HJ, and LX designed the experiment. Collections of flax germplasm resources were performed by LX and DG. DG, HJ, WY, LY, JY, YW, QY, JC, YG, LD, and HL collected data. DG and HJ analyzed data and wrote the manuscript. All authors read and approved the final manuscript.

## Funding

The research was funded by National Natural Science Foundation of China (31160056/C020408) and High Technology Project of Xinjiang Uygur Autonomous Region in China (201517108).

## Conflict of Interest

The authors declare that the research was conducted in the absence of any commercial or financial relationships that could be construed as a potential conflict of interest.
