# Supplementary material for: Resequencing 200 Flax Cultivated Accessions Identifies Candidate Genes Related to Seed Size and Weight and Reveals Signatures of Artificial Selection
Source: Front Plant Sci. 2020 Jan 16;10:1682. doi: 10.3389/fpls.2019.01682 (PMC6976528; doi:10.3389/fpls.2019.01682)
Supplement: Supplementary file 1 [file DataSheet_1.pdf]

Supplementary Material

Resequencing 200 Flax Cultivated Accessions Identifies Candidate Genes Related to Seed Size and Weight and Reveals Signatures of Artificial Selection

Dongliang Guo[1†], Haixia Jiang [1†], Wenliang Yan[2], Liangjie Yang[3], Jiali Ye[1], Yue Wang[1], Qingcheng Yan[1], Jiaxun Chen[1], Yanfang Gao[1], Lepeng Duan[1]，Huiqing Liu[1] and Liqiong Xie [1*]

[*]*Correspondence: Liqiong Xie picea@sina.com.*

[†]*These authors have contributed equally to this work.*

Supplementary Figure S1. The high-density genomic variation map spanned 15 chromosomes and contained 674,074 high-quality SNPs.

Supplementary Figure S2. Graphical phenotype of 7 major agronomic traits.

Supplementary Figure S3. Pearson's correlation coefficients between seed length (SL), seed width (SW) and 1000-seed weight (1000-SW).

Supplementary Figure S4. Genome-wide association study (GWAS) for seed size and 1000-seed weight in 2016DL.

Supplementary Figure S5. Genome-wide association study (GWAS) for seed size and 1000-seed weight in 2017UR.

Supplementary Figure S6. Genome-wide association study (GWAS) for seed size and 1000-seed weight in 2019UR.

Supplementary Figure S7. Genome-wide association study (GWAS) for seed size and 1000-seed weight in 2019YL.

Supplementary Figure S8. Boxplots for SL and SW based on the haplotypes (Hap.) for *Lus10036372*.

Supplementary Figure S9. GWAS for seed size and 1000-seed weigh and candidate genes were obtained in the peak region on chromosome 9.

Supplementary Figure S10. Boxplots for SL, SW and 1000-SW based on the haplotypes (Hap.) for *Lus10008949*.

Supplementary Figure S11. Boxplots for SL and SW based on *Lus10043126* function.

Supplementary Figure S12. Boxplots for seed size and 1000-seed weight among oil, OF and fiber flax under four environments.

Supplementary Figure S13. Comparison of nucleotide diversity (π) between landrace and improved oil flax cultivars.

Supplementary Figure S14. Pyramiding of large-seed alleles in flax varieties.

Table S1. 200 cultivars used in the study.

Table S2. Classification and annotation of SNPs in candidate regions of the peak on chromosome 11.

Table S3. Candidate gene homologs and functional annotation.

Table S4. Classification and annotation of SNPs in candidate regions of the peak on chromosome 9.

Table S5. Classification and annotation of SNPs in candidate regions of the peak on chromosome 12.

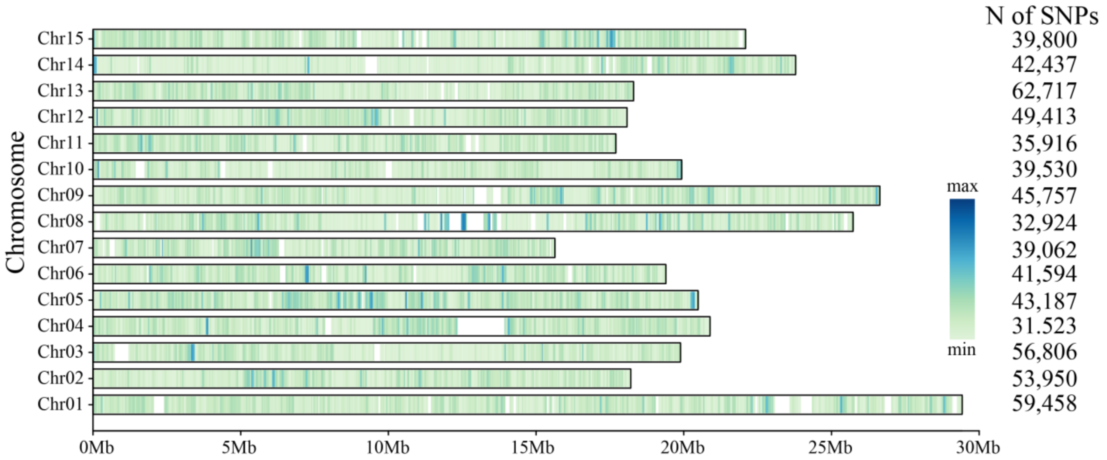

Supplementary Figure S1. The high-density genomic variation map spanned 15 chromosomes and contained 674,074 high-quality SNPs (coverage depth ≥ 3, missing rates < 0.2 and MAF ≥ 0.05).

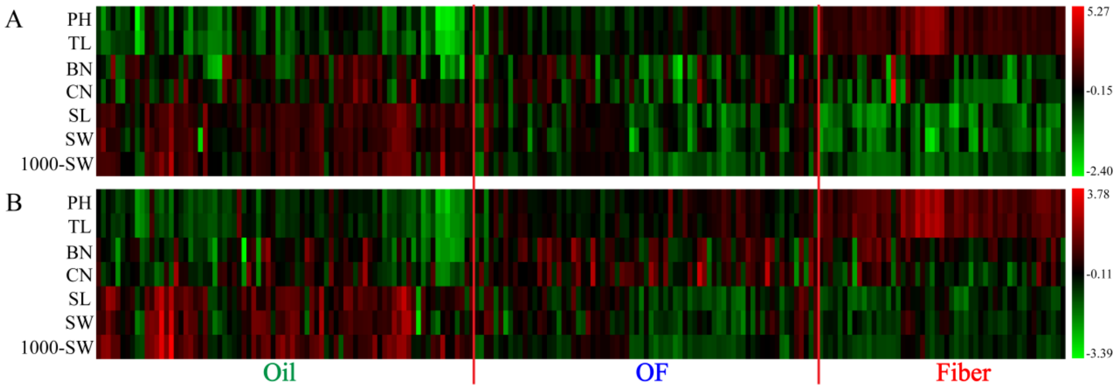

Supplementary Figure S2. Graphical phenotype of 7 major agronomic traits. **(A)** 2019UR. **(B)** 2019YL.

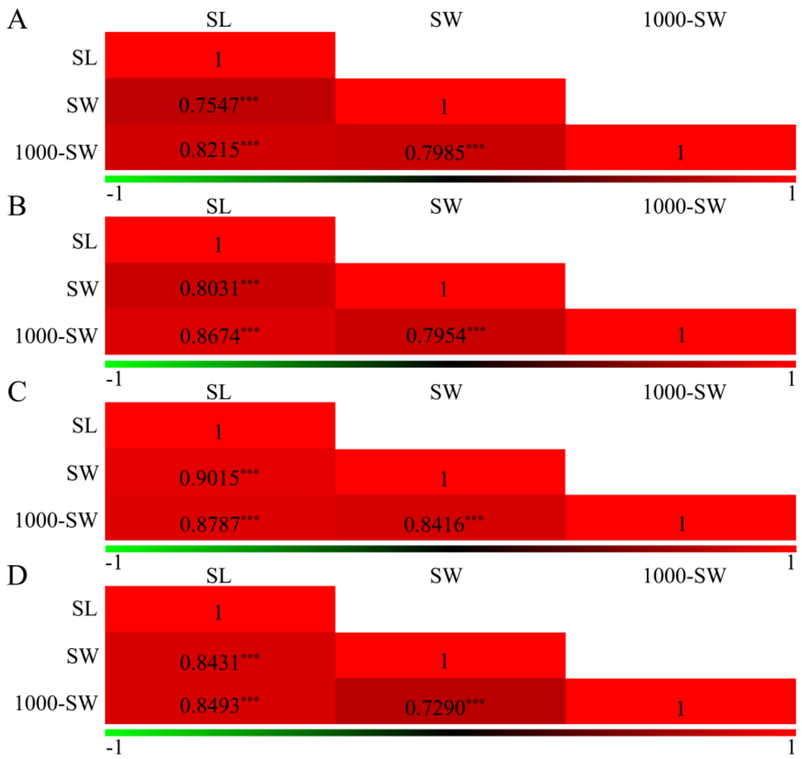

Supplementary Figure S3. Pearson's correlation coefficients between seed length (SL), seed width (SW) and 1000-seed weight (1000-SW). **(A)** 2016DL. **(B)** 2017UR. **(C)** 2019UR. **(D)** 2019YL; Significance level: $^{***}P$<0.001.

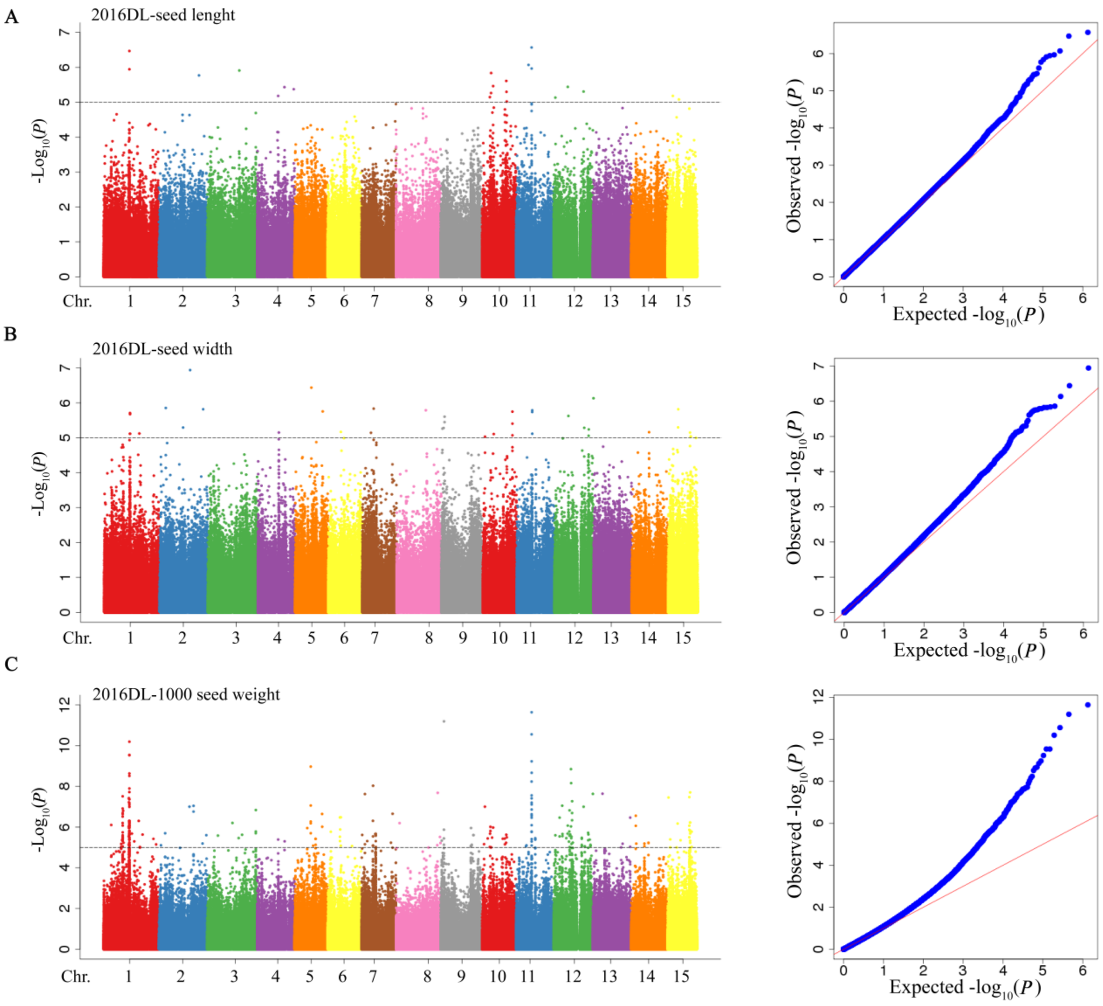

Supplementary Figure S4. Genome-wide association study (GWAS) for seed size and 1000-seed weight in 2016DL. **(A-C)** Manhattan plots with the matching QQ plots are shown for SL, SW and 1000-SW. **(A)** SL. **(B)** SW. **(C)** 1000-SW.

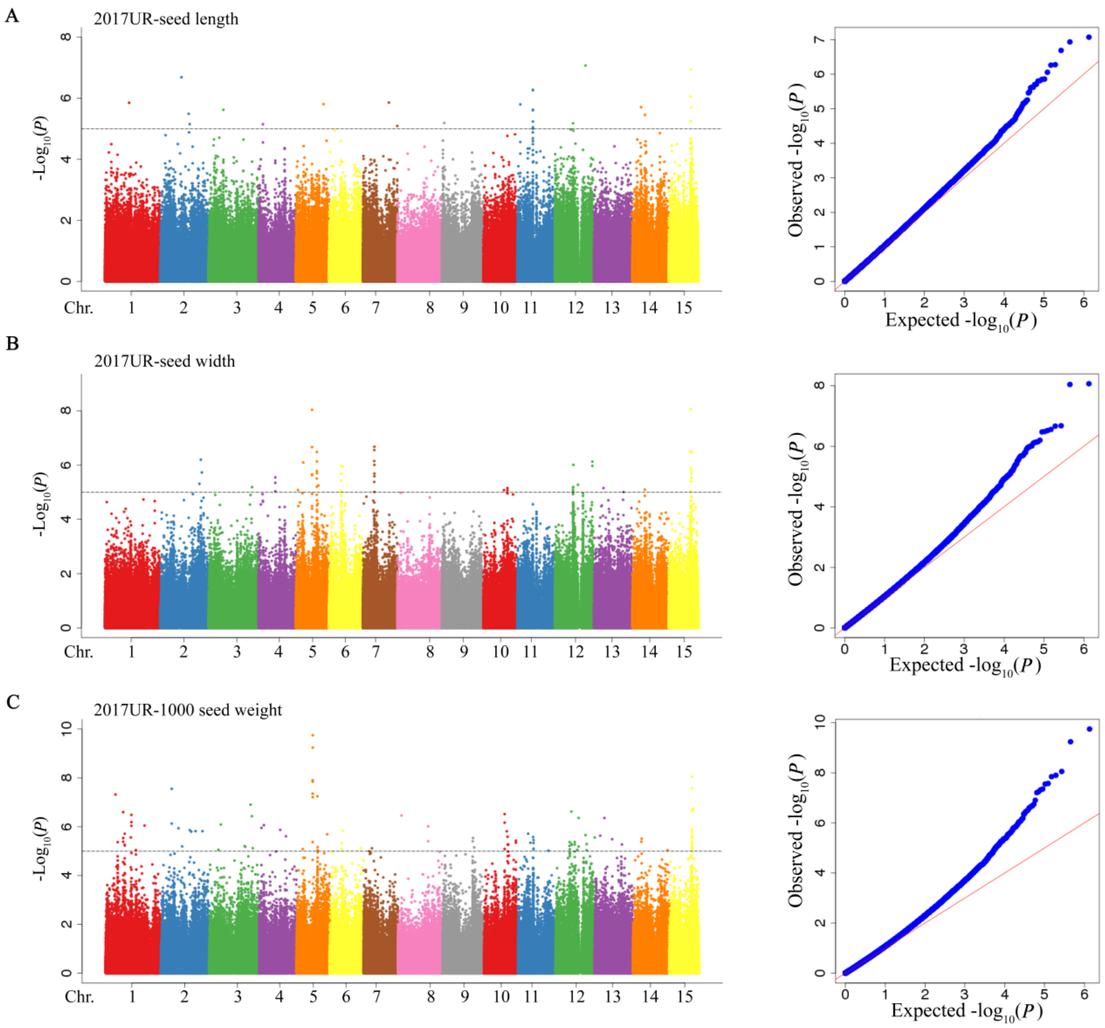

Supplementary Figure S5. Genome-wide association study (GWAS) for seed size and 1000-seed weight in 2017UR. **(A-C)** Manhattan plots with the matching QQ plots are shown for SL, SW and 1000-SW. **(A)** SL. **(B)** SW. **(C)** 1000-SW.

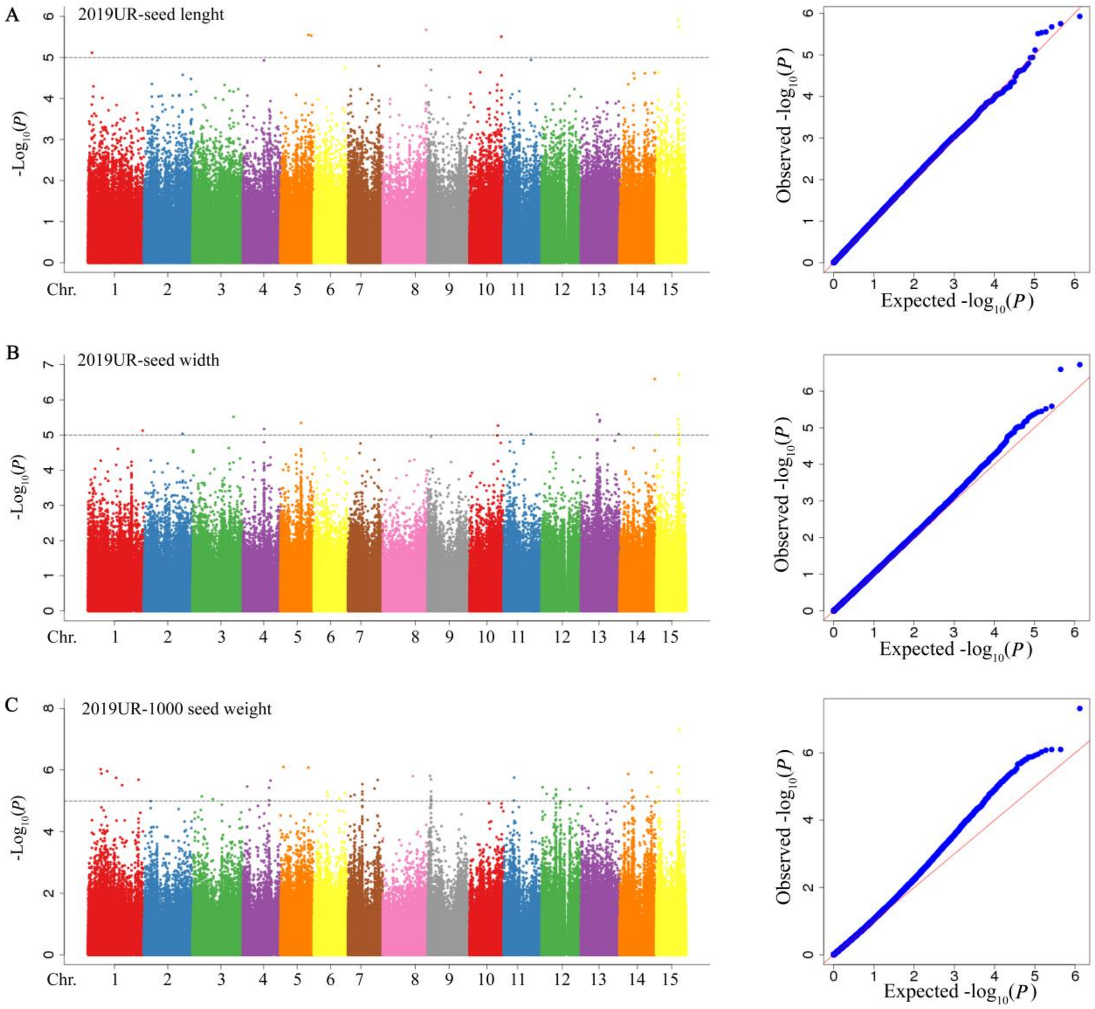

Supplementary Figure S6. Genome-wide association study (GWAS) for seed size and 1000-seed weight in 2019UR. **(A-C)** Manhattan plots with the matching QQ plots are shown for SL, SW and 1000-SW. **(A)** SL. **(B)** SW. **(C)** 1000-SW.

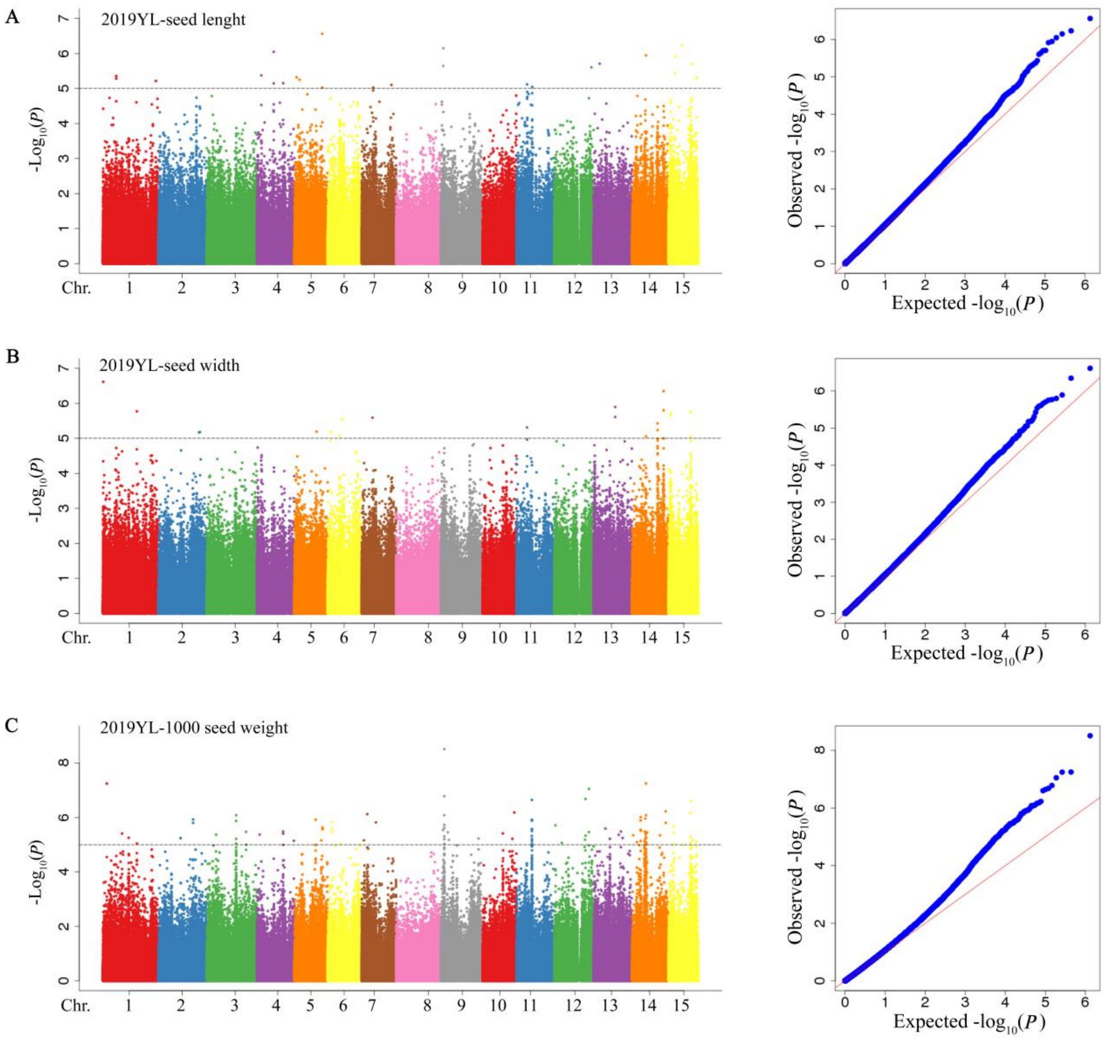

Supplementary Figure S7. Genome-wide association study (GWAS) for seed size and 1000-seed weight in 2019YL. **(A-C)** Manhattan plots with the matching QQ plots are shown for SL, SW and 1000-SW. **(A)** SL. **(B)** SW. **(C)** 1000-SW.

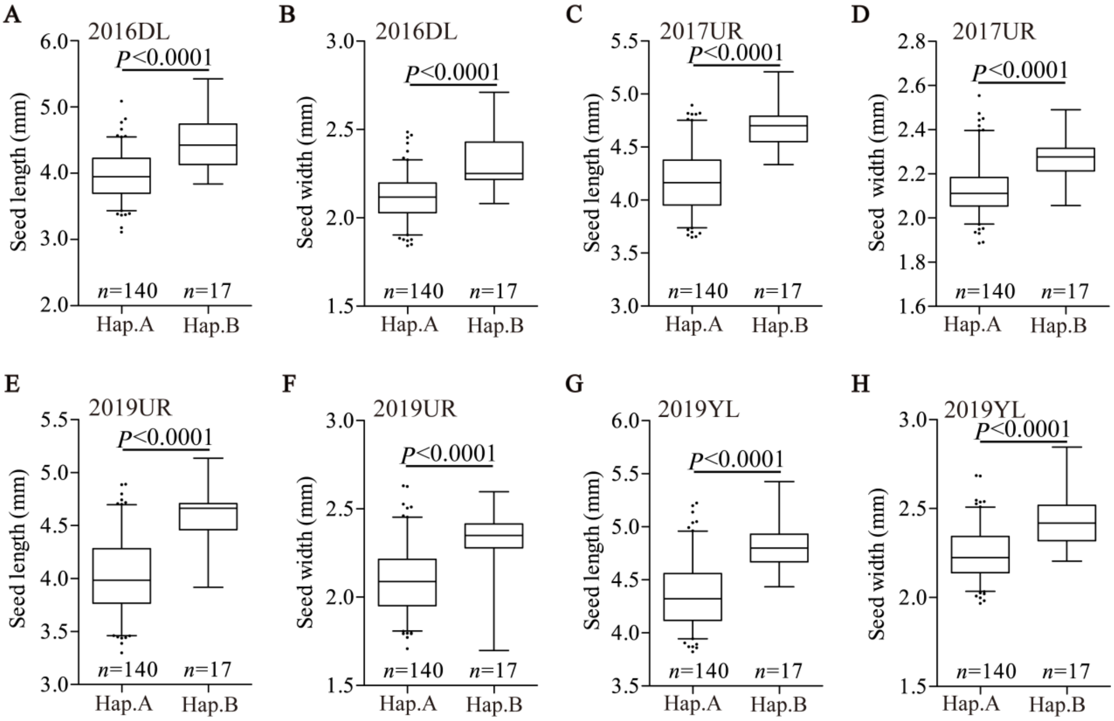

Supplementary Figure S8. Boxplots for SL and SW based on the haplotypes (Hap.) for *Lus10036372*. **(A, B)** 2016DL. **(C, D)** 2017UR. **(E, F)** 2019UR. **(G, H)** 2019YL. The difference between haplotypes was analyzed by non-parametric *t* tests.

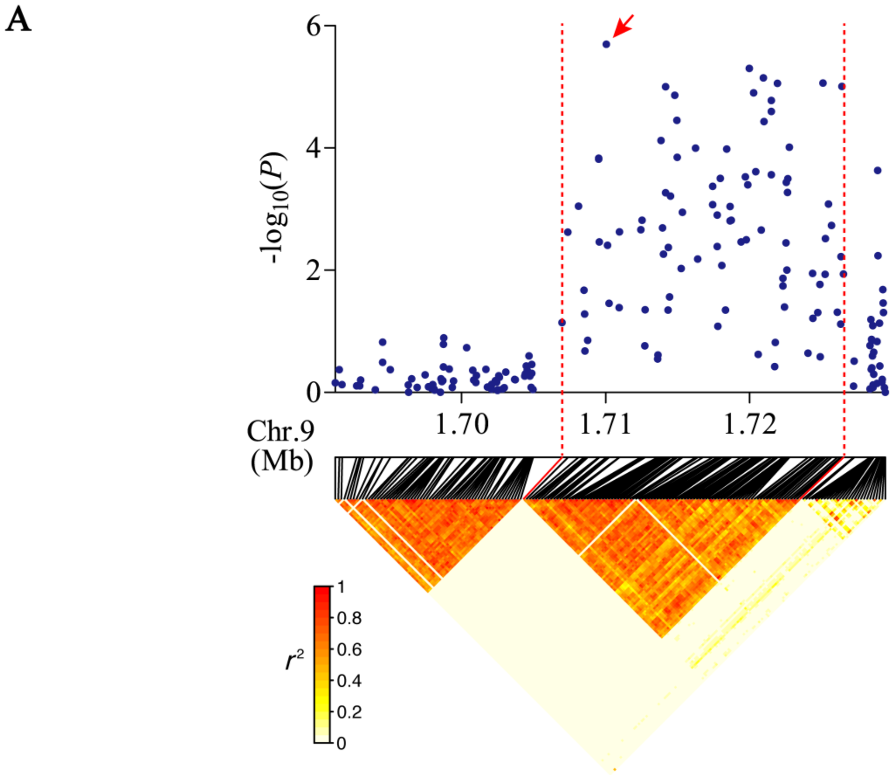

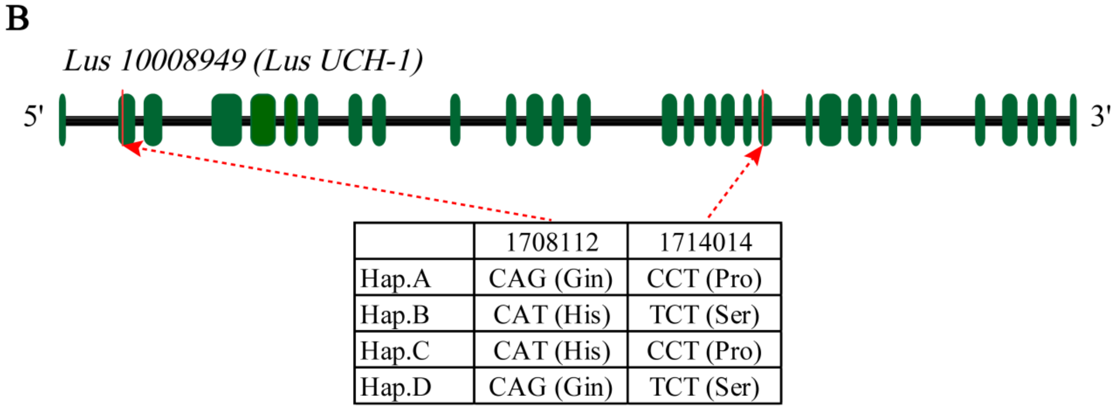

	1708112	1714014
Hap.A	CAG (Gin)	CCT (Pro)
Hap.B	CAT (His)	TCT (Ser)
Hap.C	CAT (His)	CCT (Pro)
Hap.D	CAG (Gin)	TCT (Ser)

Supplementary Figure S9. GWAS for seed size and 1000-seed weigh and candidate genes were obtained in the peak region on chromosome 9. **(A)** Local manhattan plot (top) and LD heatmap (bottom) surrounding the peak on chromosome 9. The red arrow indicates a significant SNP in candidate gene *Lus10008949*. **(B)** Exon-intron structure of *Lus10008949* and haplotypes in that gene.

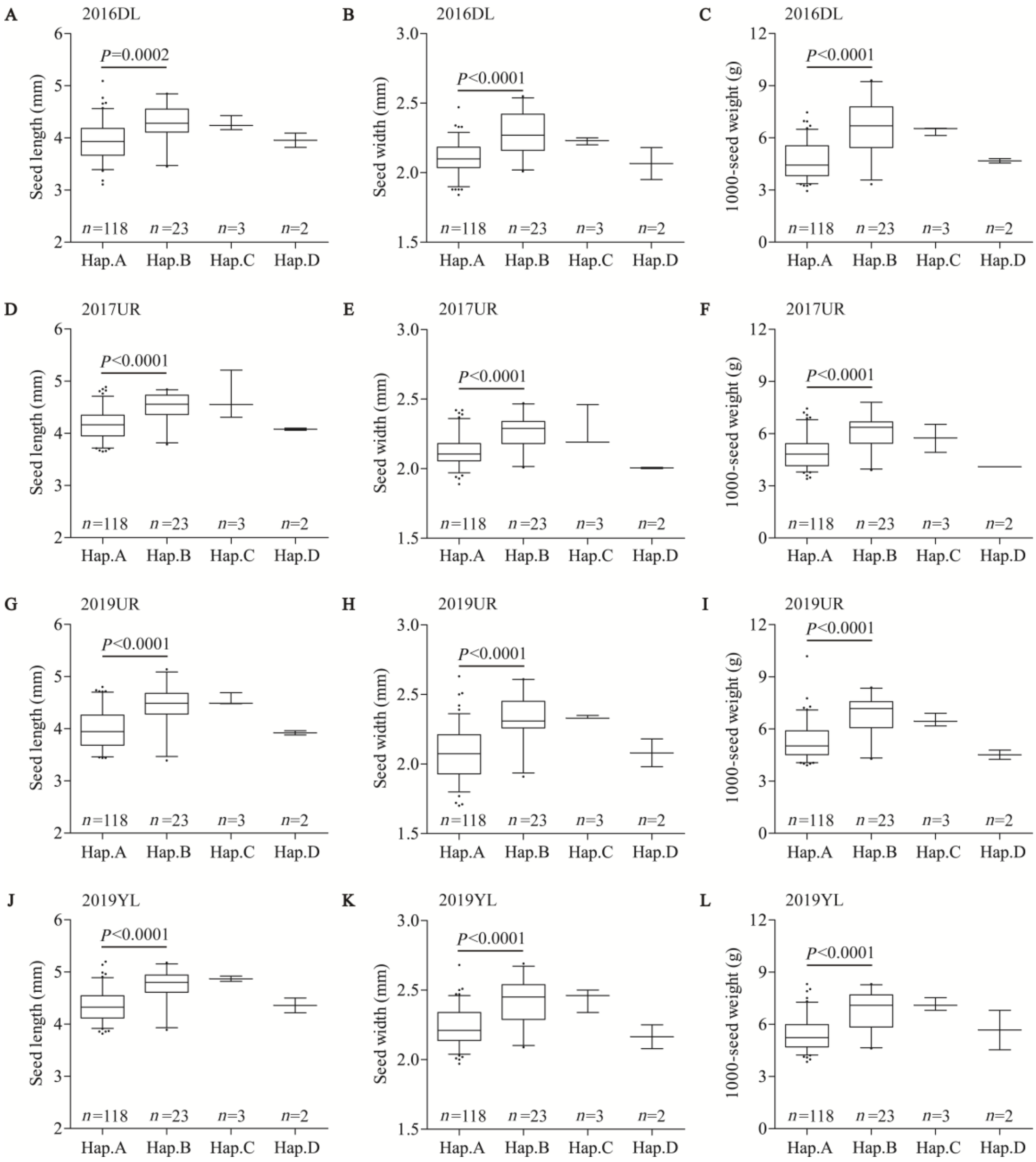

Supplementary Figure S10. Boxplots for SL, SW and 1000-SW based on the haplotypes (Hap.) for *Lus10008949*. **(A-C)** 2016DL. **(D-F)** 2017UR. **(G-I)** 2019UR. **(J-L)** 2019YL. The difference between haplotypes was analyzed by non-parametric *t* tests.

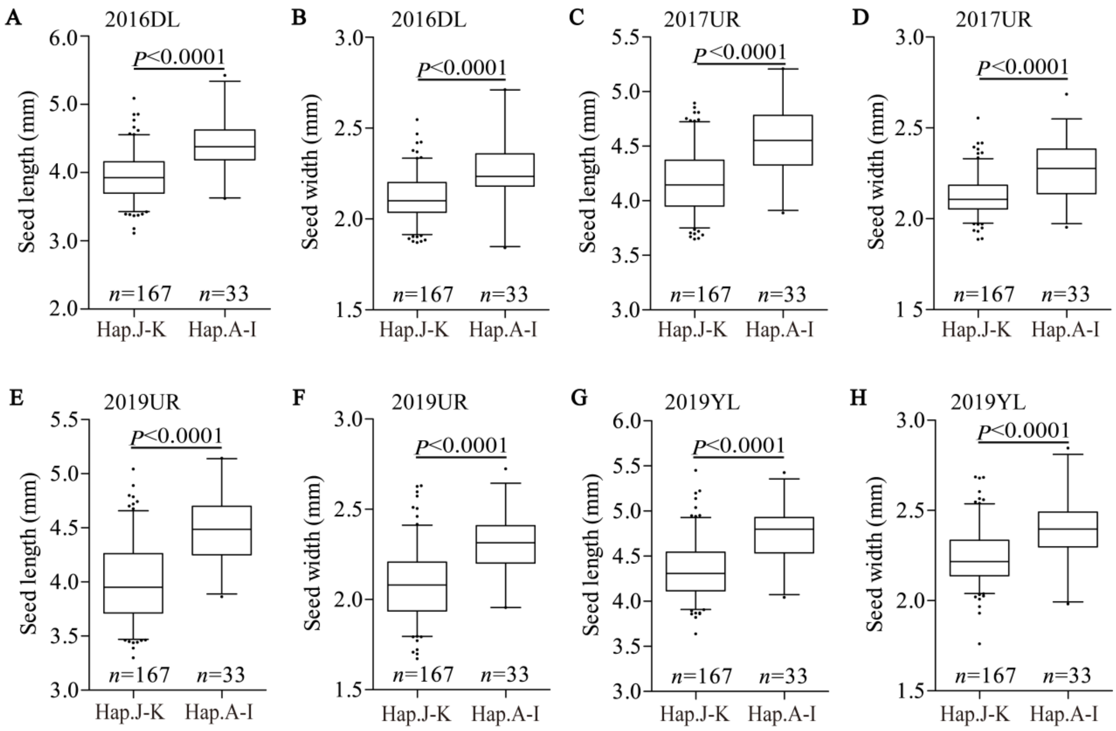

Supplementary Figure S11. Boxplots for SL and SW based on *Lus10043126* function. as follows: haplotypes A-I (functional alleles) and haplotypes j-K (nonfunctional alleles). **(A, B)** 2016DL. **(C, D)** 2017UR. **(E, F)** 2019UR. **(G, H)** 2019YL. The difference between haplotypes was analyzed by non-parametric *t* tests.

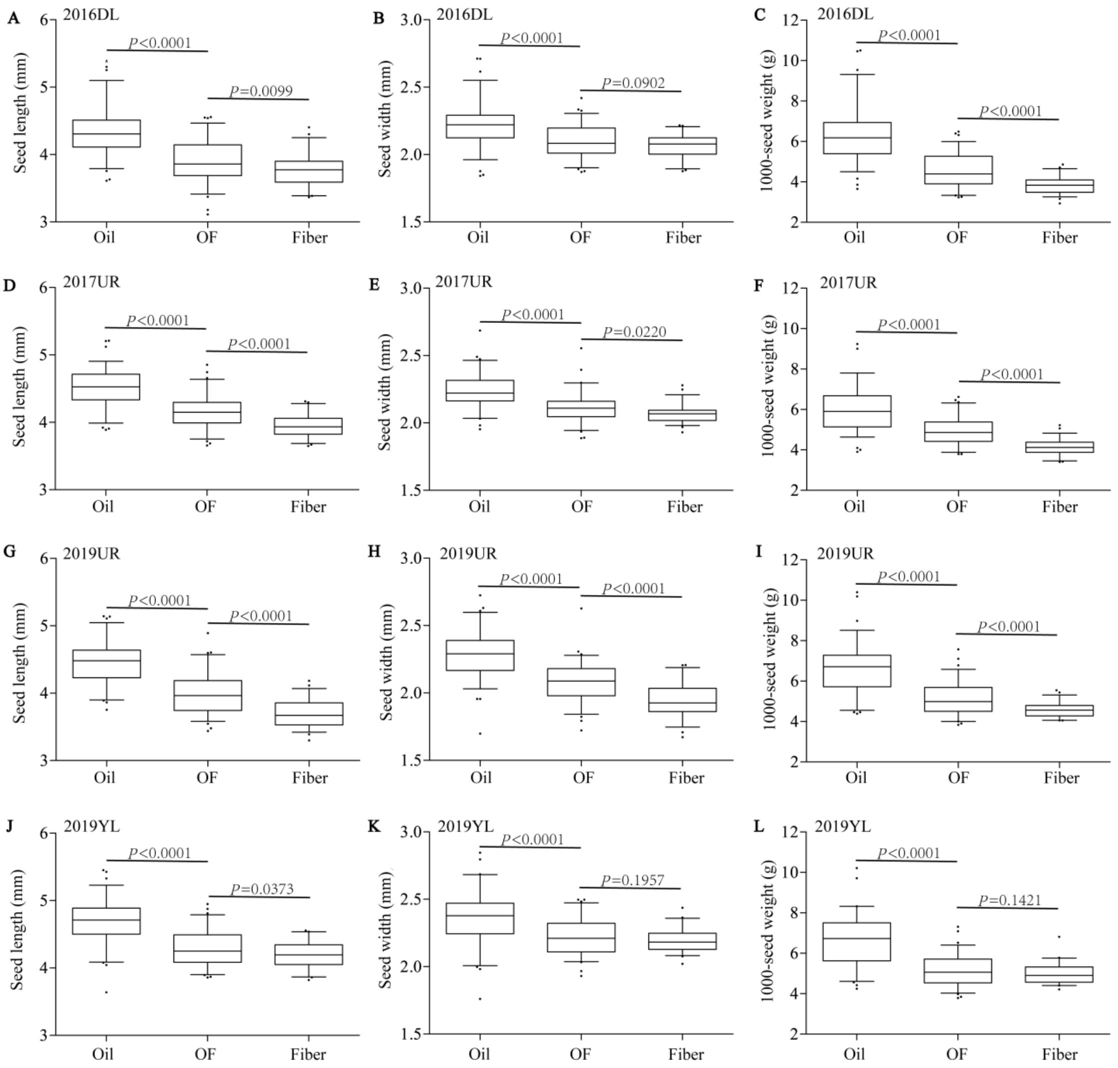

Supplementary Figure S12. Boxplots for seed size and 1000-seed weight among oil, OF and fiber flax under four environments. **(A-C)** 2016DL. **(D-F)** 2017UR. **(G-I)** 2019UR. **(J-L)** 2019YL. The traits of the three groups were compared using *t* tests.

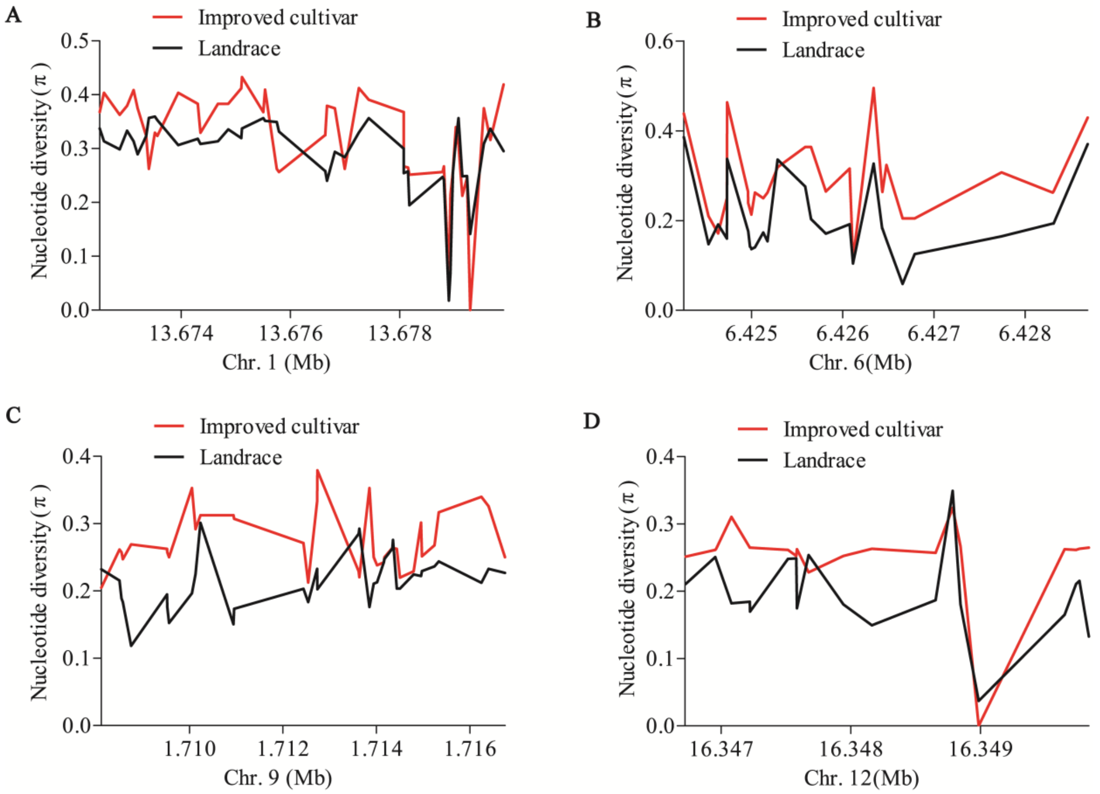

Supplementary Figure S13. Comparison of nucleotide diversity (π) between landrace and improved oil flax cultivars. **(A-D)** Local nucleotide diversity (π) was distributed around *Lus10008438* **(A)**, *Lus10017775* **(B)**, *Lus10008949* **(C)** and *Lus10043126* **(D)** among landrace (black) and improved oil flax (red).

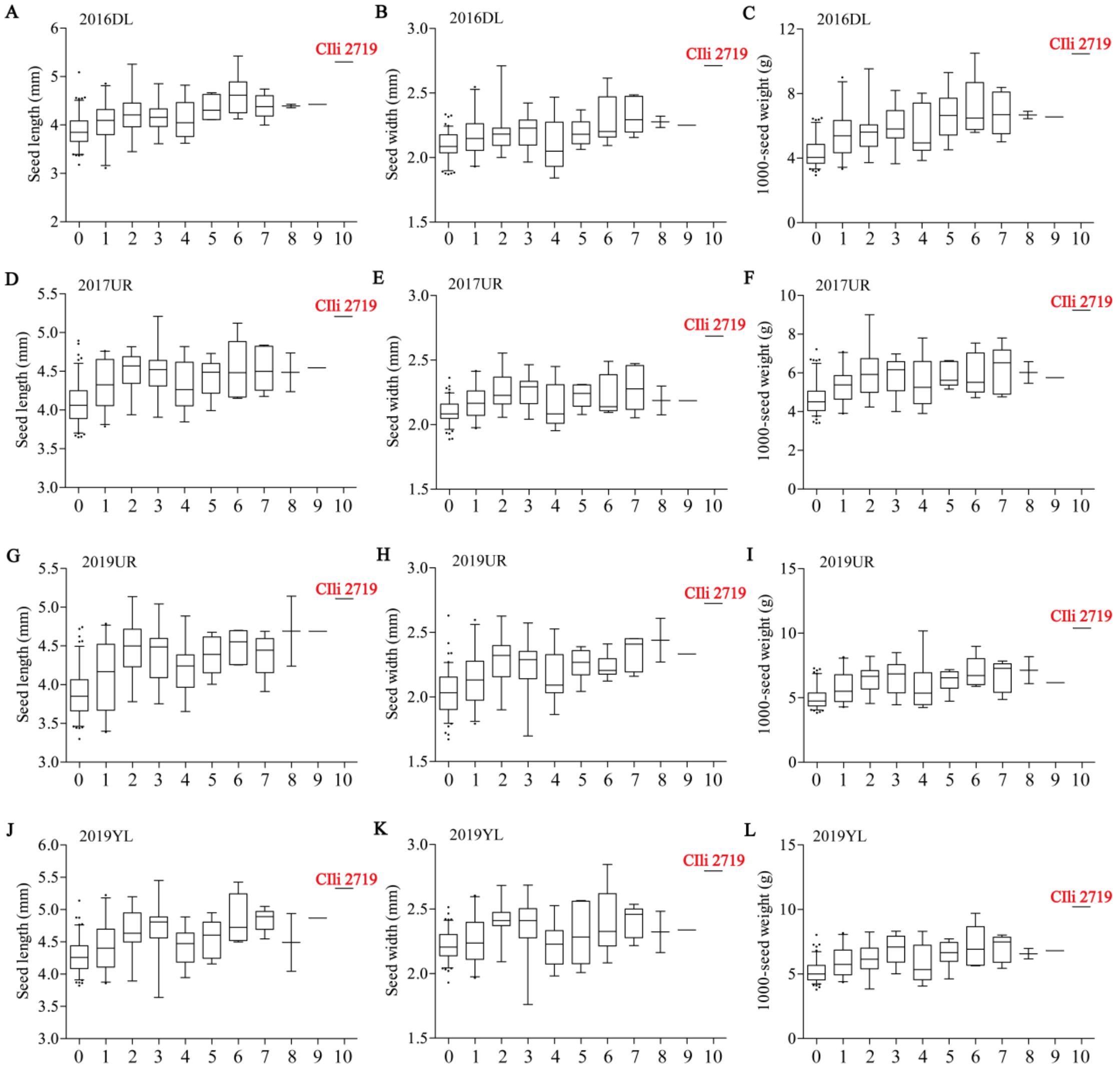

Supplementary Figure S14. Pyramiding of large-seed alleles in flax varieties. The varieties accumulated the large-seed haplotypes generally shows longer seed length and width and larger 1000-seed weight. **(A-C)** 2016DL. **(D-F)** 2017UR. **(G-I)** 2019UR. **(J-L)** 2019YL.

Table S1. 200 cultivars used in the study.

Serial number	Variety name	Origin
C1	Atalante	France
C2	Noralta	Canada
C3	Novelty	Canada
C4	Res. x Hoshangabad (C.I. 19 x C.I.140)	United States
C5	Linota	United States
C6	Sel. of N.D.R. 114	United States
C7	Dehiscent	Unknown
C8	Noname	United States
C9	Tammes Type 5	United States; Netherlands
C10	Sel. 176 x (19 x 112)	United States
C11	Lusatia	United States; Germany
C12	Hollandia	United States; Netherlands
C13	Artemida	Lithuania
C14	A-93	Russian Federation
C15	L. 270-68	Russian Federation
C16	VNIIL-180	Russian Federation
C17	VNIIL-1104	Russian Federation
C18	VNIIL-519	Russian Federation
C19	VNIIL-918	Russian Federation
C20	VNIIL-725	Russian Federation
C21	VNIIL-3531	Russian Federation
C22	Evelin	Unknown
C23	Laura	Unknown
C24	Hermes	France
C25	Ariane	France
C26	Raisa	Netherlands
C27	Escalina	Netherlands
C28	Marina	Netherlands
C29	Korostenskij 3	Ukraine
C30	Natasja	Netherlands
C31	Domtar Selection	Canada
C32	De metcha 1-3-6 Vilm	United States; Ethiopia
C33	Tammes Type 2	United States; Netherlands
C34	Tammes Type 12	United States; Netherlands
C35	Buda 80	United States
C36	Atlas (fiber)	United States; Sweden
C37	Z 11637	United States; Netherlands
C38	Mapun	United States; Hungary
C39	Erythree	United States; France
C40	Common	United States
C41	411704 Fiber	United States

C42	Tammes Pale Blue	United States
C43	Belinka	Netherlands
C44	1270	Russian Federation
C45	Svetoch mutation	Russian Federation
C46	L-93-2	China; Russian Federation
C47	L-8709-5-10	China; Russian Federation
C48	L-140-16	Russian Federation
C49	Torzhokij 4	Russian Federation
C50	Novotorzhskij	Russian Federation
C51	Aleksim	Russian Federation
C52	AR-2	Russian Federation
C53	G-1781-4-18	Russian Federation
C54	B-14	Lithuania
C55	L-1120	Russian Federation
C56	G-1847-4-1	Russian Federation
C57	Verchnevolzhkij	Russian Federation
C58	Belochka	Russian Federation
C59	VNIIL-5520	China; Russian Federation
C60	VNIIL-742	Russian Federation
C61	VNIIL-776	Russian Federation
C62	VNIIL-409	Russian Federation
C63	VNIIL-492	Russian Federation
C64	TR 35141	Russian Federation
C65	Tajga	France
C66	Line 548-01	Unknown
C67	Line 629-01	Unknown
C68	Line 657-01	Unknown
C69	Pskovski 2976	Unknown
C70	G 2063-5-10	Unknown
C71	L-500004-2-84	Unknown
C72	L-60016-3-87	Unknown
C73	Mures	Romania
C74	L-41	Russian Federation
C75	Concurent	Russian Federation
C76	China 1	China
C77	China 2	China
C78	China 4	China
C79	China 6	China
C80	Viking	France
C81	Sel. C.I. 19-47 Pale Blue	United States
C82	No. Dak. No. 40,013	United States
C83	Sel. of Minn. 281	United States
C84	VNIIL-6148	Russian Federation
C85	Macbeth	Canada

C86	Crepitam Tabor	United States; Hungary
C87	CDC Bethune-14	Germany
C88	Diane	Netherlands
C89	K-1339	Russian Federation
C90	Argos	France
C91	NOVA ROSSISK SELECTI	United States
C92	RABA 0189	United States; Poland
C93	TAMMES TYPE-XJ	United States; Netherlands
C94	XLB	Hungary
C95	Arcata	France
C96	38	Morocco
C97	CIli 452	Zimbabwe
C98	CIli 642	Russian Federation
C99	CIli 1407	Turkey
C100	CIli 1464	United States
C101	CIli 1490	India
C102	CIli 1519	Japan
C103	BELADI Y 6903	Australia
C104	URUGUAY 36/48	Uruguay
C105	CIli 1617	Afghanistan
C106	CIli 1679	Serbia
C107	Q9903	Spain
C108	Peynau	South Korea
C109	CIli 1918	Spain
C110	CIli 1919	Iran
C111	CIli 1924	Egypt
C112	CIli 2038	Iran
C113	CIli 2047	Turkey
C114	ALSEE	India
C115	MESSENIAS	Greece
C116	OLEIFERA	Poland
C117	41	India
C118	MARTIN	Hungary
C119	DIADEM	Hungary
C120	Q0415	Belgium
C121	62/125-4	India
C122	TARAGUI	Argentina
C123	TIMBU	Argentina
C124	HINDI	Czech Republic
C125	GRAND TURKISTAN 4	Russian Federation
C126	CYPRUS	Cyprus
C127	MALABRIGO 1.7	Uruguay
C128	Olayozon	Romania
C129	Tverta	Romania

C130	ARANDAS	Mexico
C131	R-111	Bolivia
C132	K-y	Romania
C133	CULBERT M3P3	United States
C134	CONCURRENT	Netherlands
C135	HERRA	Netherlands
C136	ROYAL	Canada
C137	CIli 897	United States
C138	CIli 908	United States
C139	CIli 946	United States
C140	REDSON	United States
C141	TOMAGAON	Iran
C142	MINVANO	Brazil
C143	BURKE	United States
C144	CIli 1185	United States
C145	CIli 1474	India
C146	UNRYU	Japan
C147	WELLS	United States
C148	CIli 1525	France
C149	RECOLTE NO 0196	Morocco
C150	RIJKI	Bulgaria
C151	CIli 1555	Australia
C152	BENVENUTO REAL	Hungary
C153	CIli 1581	Indonesia
C154	QUERANDI	Argentina
C155	CIli 1596	India
C156	LUOMAL MAATIAIS YELLOW	Finland
C157	LINAZA	Costa Rica
C158	BETA 201	Hungary
C159	CIli 1749	United States
C160	CIli 1819	United States
C161	Longya10	China
C162	CIli 1832	India
C163	BASIN	India
C164	N 39/B LA PLATA	Hungary
C165	CREE	Canada
C166	CIli 1966	Pakistan
C167	CIli 1976	Germany
C168	CIli 2197	India
C169	CIli 2225	India
C170	BERNBURGER OEL FASERLEIN	Germany
C171	TEXTILAK	Czech Republic
C172	CIli 2264	Canada
C173	CIli 2473	India

C174	CIli 2575	Australia
C175	CIli 2719	France
C176	CIli 2734	France
C177	STENDEZ	France
C178	WALSH	United States
C179	VICTORY	United States
C180	DOLGUNETZ	Russian Federation
C181	CIli 1676	Ethiopia
C182	CIli 1683	Monaco
C183	CIli 1689	Morocco
C184	CIli 1761	United States
C185	CIli 1777	United States
C186	WADA	United States
C187	PERCELLO	United States
C188	LIRAL PRINCE	Canada
C189	CASCADE	United States
C190	MARSIC	Italy
C191	CIli 2802	Canada
C192	ANTARES	France
C193	HINU	New Zealand
C194	CHANA	Argentina
C195	MAROS	Romania
C196	H2	Romania
C197	York	United States
C198	TJK04-20	Tajikistan
C199	TJK04-72	Tajikistan
C200	TJK04-348	Tajikistan

Table S2. Classification and annotation of SNPs in candidate regions of the peak on chromosome 11.

	**Chr.**	**Position**	**$-\log_{10}P$**	**Scaffold**	**Location**	**Gene ID**	**Region**	**Annotation**
Group I (9 SNPs in 4 genes)	11	8148722	11.64	scaffold57	339477	*Lus10036373*	Exons	ARID/BRIGHT DNA-binding domain protein
	11	8150087	7.41	scaffold57	338112	*Lus10036373*	Intron	ARID/BRIGHT DNA-binding domain protein
	11	8152763	10.55	scaffold57	335436	*Lus10036372*	Promoter	Ubiquitin-conjugating enzyme E2
	11	8154007	5.1	scaffold57	334192	*Lus10036372*	Promoter	Ubiquitin-conjugating enzyme E2
	11	8157581	7.2	scaffold57	330618	*Lus10036371*	Exons	SNF2 family N-terminal domain containing protein
	11	8157710	6.46	scaffold57	330489	*Lus10036371*	Exons	SNF2 family N-terminal domain containing protein
	11	8162032	7.11	scaffold57	326167	*Lus10036371*	Intron	SNF2 family N-terminal domain containing protein
	11	8164409	7.56	scaffold57	323790	*Lus10036371*	Exons	SNF2 family N-terminal domain containing protein
	11	8174330	6.56	scaffold57	313869	*Lus10036370*	Intron	Cysteine synthase
Group II (11 SNPs)	11	8151525	6.63	scaffold57	336674			
	11	8152152	8.24	scaffold57	336047			
	11	8156470	6.09	scaffold57	331729			
	11	8157282	5.77	scaffold57	330917			
	11	8157283	5.77	scaffold57	330916			
	11	8157296	5.77	scaffold57	330903			
	11	8157426	5.29	scaffold57	330773			
	11	8157465	9.23	scaffold57	330734			
	11	8172132	6.86	scaffold57	316067			
	11	8172170	8.67	scaffold57	316029			
	11	8172338	5.41	scaffold57	315861			
Group III (55 SNPs)	11	8148722-8176826						

Table S3. Candidate gene homologs and functional annotation.

**Candidate genes**	**Homologous gene**	**Species**	**Identity (%)**	**E-Value**	**Annotation**
Lus10008438	*AT3G44050*	Arabidopsis	53.22	0	Kinesin family member C1
Lus10029035	*LOC_Os06g43480*	Rice	38.17	1.2E-100	Cytochrome P450
Lus10034378	*AT2G09990*	Arabidopsis	85.23	1.7E-89	Ribosomal protein S9
Lus10011772	*AT5G25880*	Arabidopsis	33.49	2.6E-106	Malate dehydrogenase
Lus10017775	*LOC_Os06g09210*	Rice	28.06	2.3E-103	Cytochrome P450
Lus10035470	*LOC_Os06g49020*	Rice	63.92	4.1E-138	26S proteasome non-ATPase regulatory subunit 14
Lus10008949	*LOC_Os11g36470*	Rice	72.23	0	Ubiquitin carboxyl-terminal hydrolase 21
Lus10036372	*AT5G05080*	Arabidopsis	15.08	1.9E-12	Ubiquitin-conjugating enzyme 22
Lus10043126	*AT1G17110*	Arabidopsis	17.2	1.4E-42	Ubiquitin-specific protease 15
Lus10000489	*AT5G04690*	Arabidopsis	20.89	4.4E-37	Ankyrin repeat family protein
Lus10013178	*AT3G14172*	Arabidopsis	23.19	4.6E-121	COP1-interacting protein-related
Lus10004079	*LOC_Os03g43510*	Rice	22.34	8.4E-17	Auxin canalization
Lus10022567	*AT5G55970*	Arabidopsis	60.44	2.5E-140	RING/U-box superfamily protein

Table S4. Classification and annotation of SNPs in candidate regions of the peak on chromosome 9.

	Chr.	Position	$–\log_{10}P$	Scaffold	Location	Gene ID	Region	Annotation
Group I (6 SNPs in 3 genes)	9	1710055	5.70	scaffold1486	167586	*Lus10008949*	Intron	Ubiquitin carboxyl-terminal hydrolase
	9	1714157	5.00	scaffold1486	171688	*Lus10008949*	Intron	Ubiquitin carboxyl-terminal hydrolase
	9	1720957	5.15	scaffold1486	178488	*Lus10008952*	Exons	Uricase
	9	1721941	5.06	scaffold1486	179472	*Lus10008952*	Intron	Uricase
	9	1725095	5.06	scaffold1486	182626	*Lus10008953*	Intron	SNF7 domain containing protein
	9	1726399	5.01	scaffold1486	183930	*Lus10008953*	Promoter	SNF7 domain containing protein
Group II (1 SNP)	9	1719979	5.30	scaffold1486	177510			
Group III (84 SNPs)	9	1706976-1726510						

Table S5. Classification and annotation of SNPs in candidate regions of the peak on chromosome 12.

	**Chr.**	**Position**	**$-\log_{10}P$**	**Scaffold**	**Location**	**Gene ID**	**Region**	**Annotation**
Group I (1SNP in 1 genes)	12	16347940	7.07093	scaffold25	1315647	*Lus10043126*	Intron	Ubiquitin carboxyl-terminal hydrolase
Group II (0 SNP)	12							
Group III (49 SNPs)	12	16343194-16355981						
